# Dietary Creatine Supplementation in Gilthead Seabream (*Sparus aurata*): Comparative Proteomics Analysis on Fish Allergens, Muscle Quality, and Liver

**DOI:** 10.3389/fphys.2018.01844

**Published:** 2018-12-21

**Authors:** Denise Schrama, Marco Cerqueira, Claúdia S. Raposo, Ana M. Rosa da Costa, Tune Wulff, Amparo Gonçalves, Carolina Camacho, Rita Colen, Flávio Fonseca, Pedro M. Rodrigues

**Affiliations:** ^1^Centro de Ciências do Mar, Universidade do Algarve, Faro, Portugal; ^2^Centro de Investigação de Química do Algarve, Universidade do Algarve, Faro, Portugal; ^3^Novo Nordisk Foundation Center for Biosustainability, Technical University of Denmark, Hørsholm, Denmark; ^4^Divisão de Aquacultura e Valorização, Instituto Português do Mar e da Atmosfera, Lisbon, Portugal; ^5^Instituto Federal de Educação, Ciência e Tecnologia do Amazonas, Manaus, Brazil

**Keywords:** creatine, aquaculture, gilthead seabream, parvalbumin, proteomics, fish nutrition, muscle quality

## Abstract

The quality of fish flesh depends on the skeletal muscle's energetic state and delaying energy depletion through diets supplementation could contribute to the preservation of muscle's quality traits and modulation of fish allergens. Food allergies represent a serious public health problem worldwide with fish being one of the top eight more allergenic foods. Parvalbumins, have been identified as the main fish allergen. In this study, we attempted to produce a low allergenic farmed fish with improved muscle quality in controlled artificial conditions by supplementing a commercial fish diet with different creatine percentages. The supplementation of fish diets with specific nutrients, aimed at reducing the expression of parvalbumin, can be considered of higher interest and beneficial in terms of food safety and human health. The effects of these supplemented diets on fish growth, physiological stress, fish muscle status, and parvalbumin modulation were investigated. Data from zootechnical parameters were used to evaluate fish growth, food conversion ratios and hepatosomatic index. Physiological stress responses were assessed by measuring cortisol releases and muscle quality analyzed by *rigor mortis* and pH. Parvalbumin, creatine, and glycogen concentrations in muscle were also determined. Comparative proteomics was used to look into changes in muscle and liver tissues at protein level. Our results suggest that the supplementation of commercial fish diets with creatine does not affect farmed fish productivity parameters, or either muscle quality. Additionally, the effect of higher concentrations of creatine supplementation revealed a minor influence in fish physiological welfare. Differences at the proteome level were detected among fish fed with different diets. Differential muscle proteins expression was identified as tropomyosins, beta enolase, and creatine kinase among others, whether in liver several proteins involved in the immune system, cellular processes, stress, and inflammation response were modulated. Regarding parvalbumin modulation, the tested creatine percentages added to the commercial diet had also no effect in the expression of this protein. The use of proteomics tools showed to be sensitive to infer about changes of the underlying molecular mechanisms regarding fish responses to external stimulus, providing a holistic and unbiased view on fish allergens and muscle quality.

## Introduction

Proteins from fish are becoming an important and healthier alternative to protein from other animals. Essential amino acids, polyunsaturated fatty acids, micronutrients and high-quality proteins are present in fish in considerable amounts (Beveridge et al., 2013; Kuehn et al., 2014). Due to the high amount of consumption, fish supplies are needed to increase rapidly (Thurstan and Roberts, 2014) and subsequently aquaculture is a fast-growing industry. Farmed fish are subjected to important standards such as food safety, fish welfare, and muscle quality (Silva et al., 2012a) with the evaluation of farmed fish quality and safety being of great concern regarding human health and acceptance by consumers (with added value for the sustainability of aquaculture industry). There are many external and internal influencing factors that affect the freshness quality of fish but texture of muscle, *rigor mortis* and pH play a critical role in the evaluation of fish quality (Wang B. et al., 2015). Nutrients or special ingredients in diets are known to influence fish quality, as well as pre-slaughter stress and post-mortem processes (Silva et al., 2012a; Wang B. et al., 2015). Information regarding the use of dietary supplements as finishing strategies to modulate post-mortem degradation of overall flesh quality criteria in seabream muscle is extremely scarce. It is known that proper slaughter techniques, can spare the muscle's energy reserves and delay post-mortem degradation processes, with positive impact on flesh quality. Recently, cutting-edge technologies such as proteomics have been emerging as a valuable tool in both aquaculture products analysis and food allergens studies (Rodrigues et al., 2012; Silva et al., 2014a; Hoffmann-Sommergruber, 2016). Proteomics provides a deeper knowledge of an organism's physiological state by comparing changes in the proteome of a tissue, fluid or cell at a given moment (Rodrigues et al., 2012; Piras et al., 2016).

Fish allergies show a point prevalence of 0.6% for all aged population in Europe and a lifetime prevalence of 2.17% (Nwaru et al., 2014), in case of children this can reach 8% worldwide (Sicherer and Sampson, 2014). In 95% of the cases, fish allergies are due to the parvalbumin protein, the major allergen in fish (Kuehn et al., 2010). Enolase, aldolase and gelatin were also identified as minor fish allergens (Kuehn et al., 2014). Parvalbumins are proteins with a low molecular weight (10–12 kDa), acidic (pH 3.5–4.5), very stable, calcium-binding (Ca^2+^) and are present in higher amount in white muscle (Van Do et al., 2005) compared to dark one. Parvalbumins are divided into two lineages, α and β, but it has been shown that the majority of fish species parvalbumins belong to the β lineage (Lindstrom et al., 1996), being considered as the more allergenic one (Griesmeier et al., 2010). Various isoforms of parvalbumin have been identified depending on fish species (Beale et al., 2009) and developmental stage (Huriaux et al., 2002).

Parvalbumin is a protein present in fish muscle, where creatine is used as a molecule to enhance bioenergetics. In vertebrates, creatine takes part in the ATP (adenosine triphosphate)/PCr (phosphocreatine) phosphate energy system, being an important substrate to increase ATP by the breakdown of PCr (Kraemer et al., 2013). The endogenous synthesis of creatine is regulated by the AGAT (L-arginine:glycine amidinotransferase) enzyme, transferring the amidino group of arginine to glycine, producing L-ornithine, and guanidinoacetic acid, which is methylated, resulting in the production of creatine (Kraemer et al., 2013). In fish, creatine has been found in white muscle both in its free and phosphorylated forms and is present in higher amounts than in mammals (Hunter, 1929; Danulat and Hochachka, 1989). It contributes to the relaxation and contraction of the muscle. Creatine use has not been studied extensively in fish nutrition, but phosphocreatine has been addressed in various experiments of fish exercise (McFarlane et al., 2001). In juvenile rainbow trout, creatine supplementation showed to contribute most to a higher endurance during a fixed velocity test without differences observed in total creatine content in muscle (McFarlane et al., 2001). Also, supplementation with creatine in zebrafish showed a significant difference in lean body mass compared to control (Domas et al., 2016). The muscle mechanism of relaxation and contraction might suffer changes if the expression of specific proteins involved in the kinetics of calcium, like parvalbumin, are modified (Gallo et al., 2008). These authors studied the effect of added creatine in the diets of rats showing a significant decrease in skeletal muscle parvalbumin content (Gallo et al., 2008). In this study the authors hypothesized that elevating the capacity for high energy phosphate shuttling through creatine loading, alleviates the need for intracellular Ca^2+^ buffering by parvalbumin and increases the efficiency of Ca^2+^ uptake by Ca^2+^-ATPases.

In this study, commercial fish diets for gilthead seabream (*Sparus aurata*) were supplemented with different percentages of creatine (2, 5, and 8%) and its effects on muscle quality, proteome and parvalbumin modulation were analyzed. The liver proteome was also studied due to its central role in the majority of key metabolic processes.

## Materials and Methods

### Fish and Rearing Conditions

For this trial, 24 gilthead seabream per tank were reared in 500 L conical tanks, with natural flow-through seawater at the Ramalhete experimental station of the University of Algarve, Faro, Portugal (from July till September). Tank triplicates were used for each tested diet. Fish with an initial body weight of 170 ± 1.4 g, were fed twice a day by hand, *ad libitum* and kept with natural temperature (23.3 ± 0.93°C, with minimum and maximum of 21.2 and 25.0°C, respectively), artificial aeration (dissolved oxygen above 5 mg L^−1^), and salinity (33.2 ± 2 %0). Tanks were exposed to natural environmental and photoperiod conditions. The experiment was performed according with the fish welfare regulations established in the EU, Council Directive 2010/63/EU and Portuguese legislation for the use of laboratory animals, permit number 0420/00/000-n.9909/11/2009.

### Experimental Diets

Fish were fed a control diet (CTRL), similar to a commercial feed (Table S1), formulated based on estimated requirements of *Sparus aurata* (Sparos, Lda., Olhão, Portugal). Three different concentrations of creatine were added to the control diet in order to get experimental diets with 2, 5, and 8% of creatine (Sparos, Lda., Olhão, Portugal). The procedures for production follow the methods described in Schrama et al. (2017) with slight modifications. The creatine was incorporated in the oil fraction in concentrations (2, 5, and 8%) according to each target formulation and applied by vacuum coating in a Pegasus vacuum mixer (PG-10VCLAB, DINNISEN, The Netherlands).

### Sampling

After 69 days of trial, all fish (72 per diet) were sampled. Twelve fish per tank were randomly picked and lethally anesthetized with MS-222 (Sigma Aldrich). Blood was immediately withdrawn from the caudal vein using heparinized syringes and liver collected and weighted for hepatosomatic index. Muscle samples were taken from the right dorsal. All samples were frozen in liquid nitrogen and stored at −80°C till further analysis. The remaining 12 fish of each tank were sampled in ice and water for fish quality measurements. Filets of 5 fish were preserved on ice for instrumental texture analysis (within 24 h after slaughtering). Four fish were maintained on ice for *rigor mortis* assessment. Three fish were used for muscle pH determinations. All fish were weight and measured.

### Cortisol Measurements

Blood samples were centrifuged at 3,000 × g for 20 min and plasma collected and frozen in liquid nitrogen and stored at −80°C till further analysis. Plasma cortisol were then determined using a commercially available ELISA kit (RE52061, IBL International), previously validated for *Sparus aurata* (Lopez-Olmeda et al., 2009) with a sensitivity of 2.5 ng ml^−1^, and intra and inter-assay coefficients of variation (CV) of 2.9 and 3.5%, respectively.

### Parvalbumin and Creatine Concentration in Muscle

Parvalbumin and creatine concentration were both determined using commercially available kits following manufacturer's instructions (Fish-Check ELISA kit, Bio-Check, UK and Creatine assay kit, Sigma Aldrich, respectively).

### Glycogen Determination in Muscle

Glycogen, a carbohydrate, is the most important energy source in post-mortem muscle and was determined only in control and creatine 8% dietary treatments using the method described by Viles and Silverman (1949). Results were expressed as μg per mg of muscle (dry weight).

### Metabolic Fingerprinting by Solid Phase Transmissive Fourier Transform Infrared (FT-IR) Spectroscopy

Liver tissue is frequently used as an index of nutritional status in fish. Therefore, liver tissues of 5 fish per tank (i.e., 15 fish per dietary treatment) from control and creatine 8% dietary treatments were lyophilized. Using an agate pestle and mortar as described by (Silva et al., 2014a), each liver sample was mixed with KBr (following a ratio of 500 mg KBr per 5 mg of sample) until homogenous. The main absorption bands were attributed to the corresponding biomolecules according to Silva et al. (2014a).

### Texture Analysis

From each raw filet (5 fish per tank) a muscle section (with skin) of ~3 × 2 × 1.2 (height) cm was taken for texture profile analysis (TPA) on a TA.XT*plus* analyzer (Stable Micro Systems, Surrey, UK) equipped with a load cell of 30 kg. The muscle pieces were compressed twice with a 50 mm diameter cylindrical metal probe (P50) at a constant speed of 2 mm/s up to 40% of the filet height. Measurements were done at room temperature (~20°C). The primary characteristics hardness, springiness, adhesiveness and cohesiveness were determined. Chewiness (secondary characteristic) was calculated as the product of hardness, cohesiveness and springiness (Hyldig and Nielsen, 2001; Careche and Barroso, 2009).

### *Rigor mortis* and pH

*Rigor mortis* is one of the indexes of fish quality and it was determined using both sides of the fish at 0, 1, 2, 4, 6, 8, 24, 48, and 72 h after slaughter, as described by Matos et al. (2010). Fish were handled carefully in order to prevent secondary effects on the development of the *rigor* state. Determination of the pH values of the fish muscle was done at 0, 1, 2, 4, 6, 8, 24, and 48 h after slaughter, using a pH meter (Eutech waterproof pH spear). At each time a new incision in the flesh of the same fish was made.

### Protein Extraction and CyDye Labeling

For a total protein extraction, muscle samples were individually homogenized with an Ultra-Turrax IKA T8 (IKA-WERG) in a DIGE buffer (7 M urea, 2 M thiourea, 4% CHAPS, 30 mM Tris, pH 8.5) containing 1 mM EDTA and 1% (v/v) protease inhibitor. Homogenates were centrifuged at 13,000 × g for 10 min at 4°C to pellet insoluble material. The resulting supernatants were quantified using Quick Start™ Bradford Protein Assay with bovine albumin as standard (Bio-Rad).

In order to simplify the protein mixture and the analysis, a fractionation approach of the muscle tissue was performed. Fractionation allows to increase the number of visualized proteins and to raise the concentration of low-abundance proteins by depletion of the highly abundant myofibrillar proteins (Silva et al., 2010, 2012a). Proteins from the sarcoplasmic fraction of the muscle were extracted using a lysis buffer (50 mM Tris-HCl, pH 7.4, 1 mM EDTA, 10 mM DTT) containing 0.5% (v/v) protease inhibitor. An Ultra-turrax IKA T8 (IKA-WERG) was used to homogenize the samples and after a 30 min settle on ice centrifugation occurred for 20 min at 11,200 × g at 4°C. The resulting supernatants were then depleted of non-protein contaminants using a ReadyPrep™ 2D Clean-up kit (Bio-Rad) and resuspended in DIGE buffer. Proteins were quantified as described before.

For DIGE minimal labeling, after pH adjustment of protein extracts to pH 8.5 by addition of 0.3 M NaOH, 50 μg of proteins were labeled with 400 pmol of fluorescent amine reactive cyanine dyes freshly dissolved in anhydrous dimethyl formamide following manufacturer's instructions (5 nmol minimal labeling kit, GE Healthcare). Three samples per dietary treatment were labeled with Cy3 and three with Cy5 to prevent confounding of an eventual “dye effect” with the biological effect we want to measure. An internal control consisting of equal quantities of protein from all samples was labeled with Cy2.

Liver samples were individually homogenized using DIGE buffer as described above for total protein extraction. The resulting supernatants were then depleted of non-protein contaminants using a ReadyPrep™ 2-D Cleanup kit (Bio-Rad) and proteins were quantified and labeled as previously described.

### Two-Dimension Gel Electrophoresis

Labeled proteins from muscle samples were first separated according to their isoelectric point on 24 cm Immobiline™ Drystrip with a pH 3–7 linear gradient (GE healthcare) as parvalbumin—the target protein—has an known acidic pH of ≈4 (Van Do et al., 2005). In case of liver samples a pH of 4–7 linear gradient has been chosen following the findings of Richard et al. (2016) in which this pH ensure the best compromise between high coverage and good protein separation. For each strip, 50 μg of protein of one sample from each dietary treatment plus 50 μg of internal standard, diluted in rehydration buffer (ReadyPrep 2-D starter kit, Bio-Rad) to a final volume of 450 μl, were loaded overnight in an IPG box (GE Healthcare) by passive rehydration. Isoelectric focusing was performed using an Ettan™ IPGphor™ 3 isoelectric focusing unit (GE Healthcare), at 20°C.

In muscle samples voltage gradually raised from 0 to 500 V over the course of 1 h, kept constant at 500 V for 1 h, then gradually raised to 1,000 V over the course of 1 h and finally gradually raised to 8,000 V over the course of 3 h, finishing with a step of 5 h 40 min at a constant voltage for a total of 60,000 V.h, with a maximum current of 75 μA per strip. In liver samples voltage gradually raised from 0 to 250 V over the course of 4 h, then gradually raised to 1,000 V over the course of 6 h and finally gradually raised to 8,000 V over the course of 3 h 40 min, finishing with a step of 3 h 20 min at a constant voltage of 8,000 V for a total of 48,000 V.h, with a maximum current of 75 μA per strip.

After separation of proteins in the first dimension, focused proteins were reduced for 15 min in 6 ml of equilibration buffer (6 M urea, 50 mM Tris-HCl, pH 8.8, 2% (w/v) SDS, 30% (v/v) glycerol) with 2% (w/v) DTT, and then alkylated for 15 min in 6 ml of equilibration buffer with 2.5% (w/v) iodoacetamide. The equilibrated strips were then placed onto 12.5% acrylamide gel cast between low fluorescence glass cassettes (EttanDALT six gel caster system, GE Healthcare), and sealed with 0.5% (w/v) agarose in 1× electrophoresis buffer [25 mM Tris, 192 mM Glycine, 0.1% (w/v) SDS] and a trace of bromophenol blue. Proteins were separated according to their molecular weight in a second dimension by SDS-PAGE, using an EttanDALT system under constant amperage of 10 mA per gel for 1 h followed by constant amperage of 40 mA per gel until the bromophenol blue dye front reached the bottom of the gels. The electrophoresis buffer was used at 1× concentration in the lower chamber and 2× concentration in the upper chamber.

### Gel Image Acquisition and Analysis

Obtained gels were scanned with a Typhoon Trio™ Variable Mode Imager (GE Healthcare) using three laser emission filters (520BP40 for Cy2, 580BP30 for Cy3, 670BP30 for Cy5) at a resolution of 100 μm. Image analysis was performed using the SameSpots™ Software (TotalLab). A filter of average normalized volume ≤ 100,000 and a spot area ≤500 was used to eliminate small impurities before analysis. Preliminary assessment of data quality was performed using Principal Component Analysis with autoscaling. 2DE gel analysis was performed following guidelines of Silva et al. (2014b).

### Protein Identification by Mass Spectrometry

Spots with a significant difference by one-way variance of significance [ANOVA (*p* < 0.05)] were manually excised from the preparative gels. Also a false discovery rate (FDR) of *q* < 0.05 was applied to minimize the number of false positives. Protein spots from the muscle samples were identified at the GIGA proteomics facility (Liège University, Liège, Belgium) while protein spots from liver samples were sent and analyzed at the Center of Biosustainability (Technical University of Denmark, Hørsholm, Denmark). At the GIGA proteomics facility trypsin in gel digestion was performed in 96 well plate format on the working station Janus (Perkin Elmer). Spots were washed twice for 5 min in 50 μl of 50 mM ammonium bicarbonate on a shaker. Wash was discarded and 50 μl of 50% acetonitrile/50 mM ammonium bicarbonate was added for 5 min on a shaker, this step was repeated once. Fifty microliter of 10 mM DTT was added per well and left on a shaker for 45 min at 56°C. The DTT was removed and 40 μl of 55 mM iodoacetamide was added and mixed for 1 min. The plate was incubated at 20°C for 1 h. Liquid was discarded and 50 μl of 50% acetonitrile/50 mM ammonium bicarbonate was added for 5 min on a shaker (twice). Wash was discarded and 50 μl of 50% acetonitrile/50 mM ammonium bicarbonate was added for 5 min on a shaker (twice). Sixty microliters of 100% acetonitrile were added for 5 min on a shaker. Liquid was discarded and the step repeated. Spots were dried for 1 h at 40°C followed by 1 h at 20°C. Trypsin was prepared in 25 mM ammonium bicarbonate (10 ng/μl) and 3 μl was added, mixed for 1 min and incubated for 1 h at 4°C followed by 4 h at 37°C. After digestion, 18 μl of 1% formic acid was added to the gel pieces and incubated for 30 min at 40°C on a shaker. PMF and MSMS analysis was performed on a MALDI-TOF-TOF-MS UltrafleXtreme (Bruker). Automatic spectra acquisition was piloted with the software Flex control™ vs. 3.4 and real time analysis by Flex analysis™ vs. 3.4 (Bruker). Search on databases were managed in real time with BioTools™ vs. 3.2 (Bruker) on the Mascot server vs. 2.2.06. Search was performed on SwissProt database restricted to Actinopterygii taxonomies with 100 ppm of mass error tolerance in MS and MSMS precursor and 0.3 Da tolerance on MSMS fragments. A second search was made with the same parameters on NCBI database restricted to Actinopterygii taxonomies.

At the Center of Biosustainability protein spots were identified by LC-MS/MS after proteins were cleaved by trypsinization. The procedures for the identification of proteins followed the protocol as described in Moreira et al. (2017). Raw data files Protein identification was obtained using the Protein Lynx Global Server (PLGS) software v2.5.3 (Water corporation) using the in-build MS^E^ search function against the databases generated from UniProt from the taxonomy Actinopterygii. In the database a list of known contaminants was added. The search parameters were trypsin as enzyme, carboxamidomethyl on cysteine as fixed modification and oxidation of methionine as partial modification while allowing one missed cleavage.

### Statistical Analysis

Statistical significance was assessed using a one-way analysis of variance [ANOVA (*p* < 0.05)] followed by a *post-hoc* Tukey (*p* < 0.05). In case of glycogen determination and FTIR analysis a Student's *T*-test was performed (*p* < 0.05). Cortisol and r*igor mortis* data were previously transformed by log and arcsine square root, respectively. Normality and homoscedasticity assumptions were previously checked using Shapiro–Wilk and Levene's tests, respectively, (*p* < 0.05). In case of cortisol analysis, a Grubbs' test was performed prior to statistical significance analysis. Results are presented in mean ± standard error of the mean (S.E.M.). All statistical analyses were performed using the R project for statistical computing (version 3.5.0) and GraphPad^®^ v6.0 for windows was used for chart building and figures layout.

## Results and Discussion

### Zootechnical Analysis

**Figure 1:**
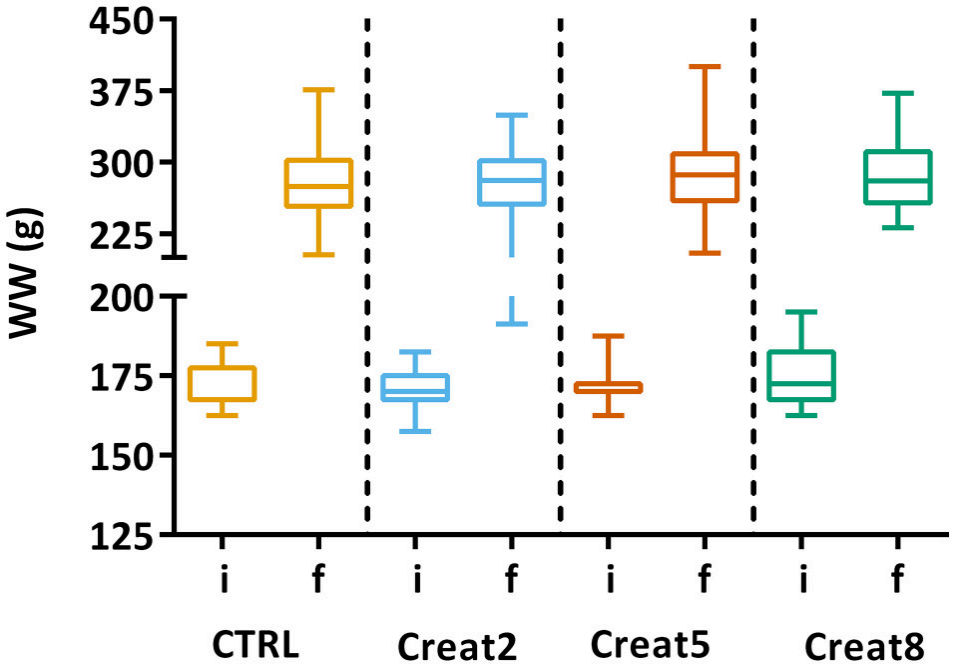
Box plots of the fish weight distributions. Plot showing the distributions of wet weight (WW) of fish for each treatment (*n* = 72) at the start of the trial (i-initial) and after 69 days (f-final). Results are shown by quartiles and the horizontal line in each box shows the median. No significant differences were observed among the treatments (one-way ANOVA, *p* > 0.05).

**Figure 2:**
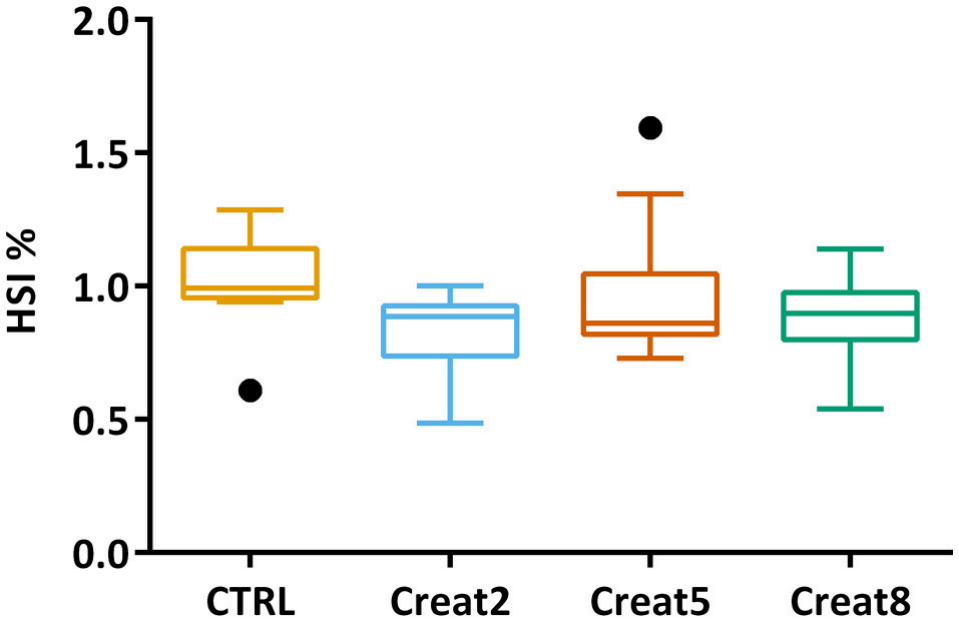
Box plot of the fish hepatosomatic index distribution. Plot showing the distributions of fish hepatosomatic index estimated from individual measurements (*n* = 15 per treatment) at the end of the trial. Results are shown by quartiles and the horizontal line in each box shows the median. No significant differences were observed among treatments (one-way ANOVA *p* > 0.05).

In this study, at a performance level, fish fed with the different tested diets, show similar growth after 69 days of trial. The box plots show the initial distribution of the fish, with a mean body weight of 170 ± 1.4 g and a final body weight of 281 ± 4.15 g (Figure 1). In Table 1, zootechnical results show the initial and final body weight (IBW and FBW, respectively), weight gain per day, specific growth rate (SGR) per day, thermal growth coefficient (TGC), feed conversion rates (FCR), and feed efficiency (FE) for all treatments without any significant differences among treatments and no mortalities were registered. Moreover, hepatosomatic index (HSI) results (Figure 2) show no significant differences among treatments suggesting that supplementation with creatine have not altered the energy reserves in these fish.

**Table 1:** Fish performance parameters.

**Diet**	**IBW (g fish^**−1**^)**	**FBW (g fish^**−1**^)**	**%IBW/day Weight Gain^a^**	**%/day SGR^b^**	**TGC^c^ (10^**−3**^ g^**1/3**^^**°**^C^**-1**^ day^**−1**^)**	**FCR^d^**	**FE^e^**
Ctrl	172 ± 3	278 ± 3	0.89 ± 0.01	0.69 ± 0.01	0.20 ± 0.001	1.66 ± 0.03	0.60 ± 0.01
Creatine2	171 ± 2	278 ± 4	0.91 ± 0.05	0.71 ± 0.03	0.20 ± 0.009	1.63 ± 0.10	0.61 ± 0.04
Creatine5	172 ± 2	285 ± 10	0.95 ± 0.06	0.73 ± 0.04	0.21 ± 0.012	1.58 ± 0.07	0.64 ± 0.03
Creatine8	175 ± 1	286 ± 12	0.91 ± 0.09	0.71 ± 0.06	0.21 ± 0.018	1.66 ± 0.08	0.60 ± 0.03

*Table with initial body weight (IBW), final body weight (FBW), weight gain per day, specific growth rate (SGR) per day, thermal growth coefficient (TGC), feed conversion rate (FCR) and feed efficiency (FE) calculated per treatment (n = 72) at the end of the trial (69 days). Values are expressed as mean ± standard deviation. Statistics by ANOVA show no significant differences (p > 0.05)*.

a Weight gain per day, calculated as [(BM_f_BM_i_) ^*^ 100]/(BM_i_^*^ t_f_), where BM_f_ and BM_i_ are the final and initial biomass, respectively, and t_f_ are the days of the trial.

b Specific growth rate, calculated as SGR (% per day) = 100 ^*^ [(Ln (FBW)–Ln (IBW)]/t_f_), where FBW and IBW are the final and initial fish body weight, respectively, and t_f_ are the days of the trial.

c *Thermal growth coefficient, calculated as TGC (10^−3^ g^1/3^ °C^−1^ day^−1^) = [(^3^√FBW−^3^√IBW)/(T x t)] x 1,000, where FBW and IBW are the final and initial fish body weight, respectively, T is the mean temperature and t are total days of the trial*.

d *Feed conversion ratio, calculated as FCR = FC/(BM_f_−BM_i_), where FC is the feed consumption and BM_f_ and BM_i_ are the final and initial biomass, respectively*.

e *Feed efficiency, calculated as FE = (BM_f_−BM_i_)/FC, where BM_f_ and BM_i_ are the final and initial biomass and FC is the feed consumption*.

Creatine supplementation does not seem to affect fish growth and the efficiency of feed is very similar between the different concentrations tested. These results are contradictory to those reported in a previous study performed in Red drum (*Sciaenops ocellatus*) for 7 weeks, where this species has benefit from dietary creatine supplementation in practical diets with improvements observed in weight gain and feed efficiency. Nevertheless, this trial was conducted under stressful conditions of low salinity which might have contributed to the differences to our outputs. Also, this fish species is produced in higher temperatures, which are related with higher intrinsic energy demands. The regulation and usage of creatine is known to be dependent on the body temperature, which in fish is dependent on the surrounding temperature (Burns and Gatlin, 2016). In rainbow trout, for instance, Borchel et al. (2014) showed differences in their gene-expression regarding acclimation temperatures lined up with creatine expression, supporting the previous statement. Regarding our study, this raises the question if creatine supplementation, has led to a higher muscle power output (to some extent) as seen in mice (Gallo et al., 2008) rather than to an effective effect on growth parameters.

### Metabolites

**Figure 3:**
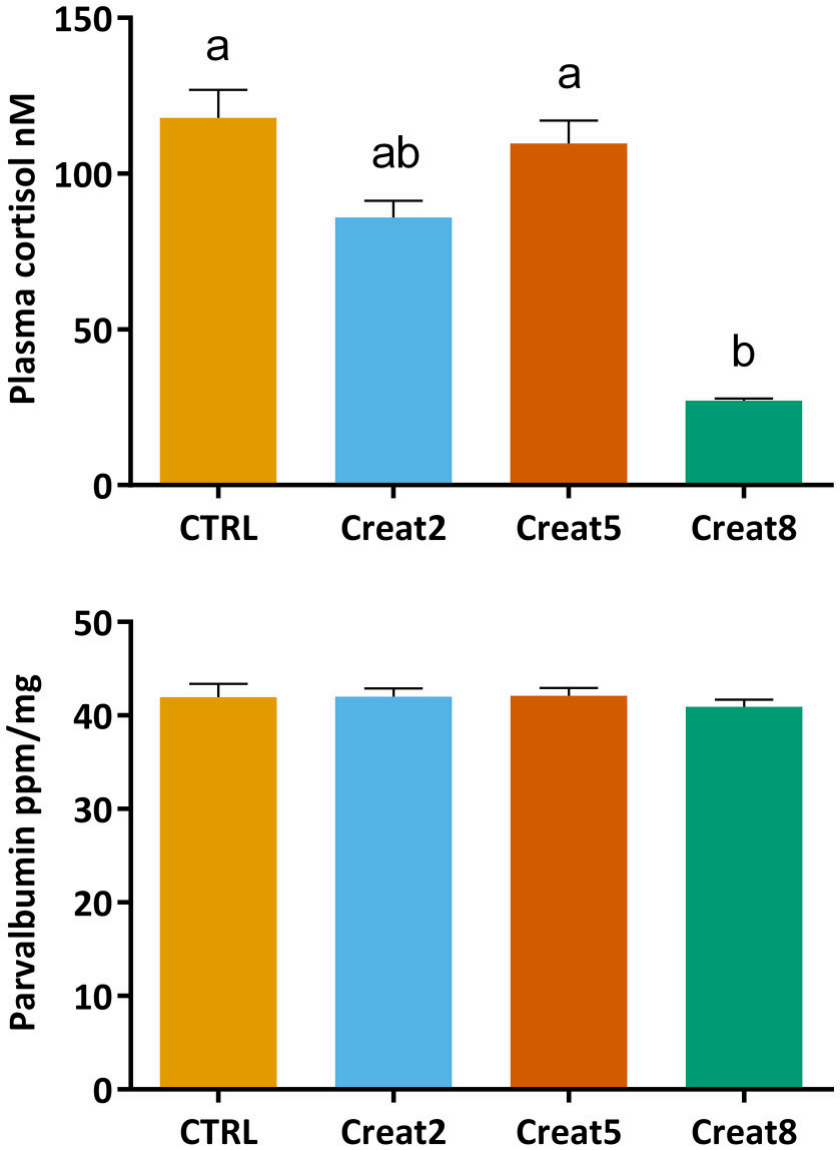
Cortisol (nM) concentration in plasma and Parvalbumin (ppm/ng) concentration in muscle of gilthead seabream after 69 days of trial. Values are means (*n* = 15) and errors bars represent standard error of the mean (SEM). Different letters represent significant differences (one-way ANOVA followed by *post-hoc* Tukey, *p* < 0.05).

To analyze the first stress barrier in fish, cortisol levels were determined using an ELISA test. Our findings show that fish fed with 8% supplementation creatine diets show significantly lower values of cortisol than fish fed with control diets and fish fed with 5% supplementation of creatine (*p* = 0.006 and *p* = 0.014, respectively; one-way ANOVA followed by *post-hoc* Tukey) (Figure 3). As mentioned above, if higher levels of creatine in the diet are related to a higher endurance capacity, as shown by McFarlane et al. (2001) with rainbow trout, one would expect fish fed creatine supplemented diets to show lower increases in plasma cortisol concentrations than fish fed a non-supplemented diet. As so, feeding periods, normally related with increases of cortisol levels, would originate less individual arousal and more ability to adjust their performance and behavior. Hence, lower cortisol levels would be detected with loading creatine diets. Lower arousal lined with reduced cortisol levels were seen by Sanchez et al. (2009) in seabream, when subjected to a random vs. schedule feeding times.

Nevertheless, there are various factors that alter cortisol levels in addition to stress which has been often reviewed [several factors have been referred, that might influence the divergent concentrations reported over similar conditions, within and between fish species (see review from Ellis et al., 2012)]. Excess cortisol levels have been associated with poor growth in goldfish, despite normal food intake (Bernier et al., 2004). In our trial, although fish show high values of cortisol, physiological stress does not seem to be affected. In fact, increasing creatine concentration, seems to decrease fish stress levels. Supporting such statement, in our study, fish almost doubled body weight during the trial with an expectable FCR (Santos et al., 2010).

Parvalbumin concentrations in fish muscle were determined using a commercially available ELISA kit which is designed for cod fish. After a blast search (blast.ncbi.nlm.nih.gov), 71% of identity with *Sparus aurata* was obtained for β-parvalbumin. Although we cannot conclude this assay to be quantitative for this species, our results show no significant differences in the parvalbumin concentration in muscle of fish fed with the different tested diets (Figure 3). We hypothesize that creatine loading would decrease the expression of parvalbumin, therefore decreasing the allergenic status of fish. According to our research outputs, creatine supplementation seems not to modulate parvalbumin expression in the muscle of gilthead seabream. These results were further validated by our comparative proteomics results and explained further down. Parvalbumin is involved in the muscular system of contraction/relaxation and plays a role in the whole fish performance. It was found that fastest sprinters release energy faster because of the release of ATP-enzyme creatine kinase (Knight, 2012). Our dissonant findings may be explained by the lack of an environmental or artificial challenge (e.g., nutritional or a stressful condition) that could increase fish activity and energy release, hence the parvalbumin concentration. Previous research regarding the expression of parvalbumin in fish has been driven mainly to determine the allergenic cross-reactivity between fish species (Van Do et al., 2005; Kuehn et al., 2010) or linked to a performance test after a stressor situation (Knight, 2012; Seebacher and Walter, 2012) which can sustain the previous statement. Nevertheless, we show the first evidences about the effect of creatine supplementation on parvalbumin modulation in non-stressed fish.

**Figure 4:**
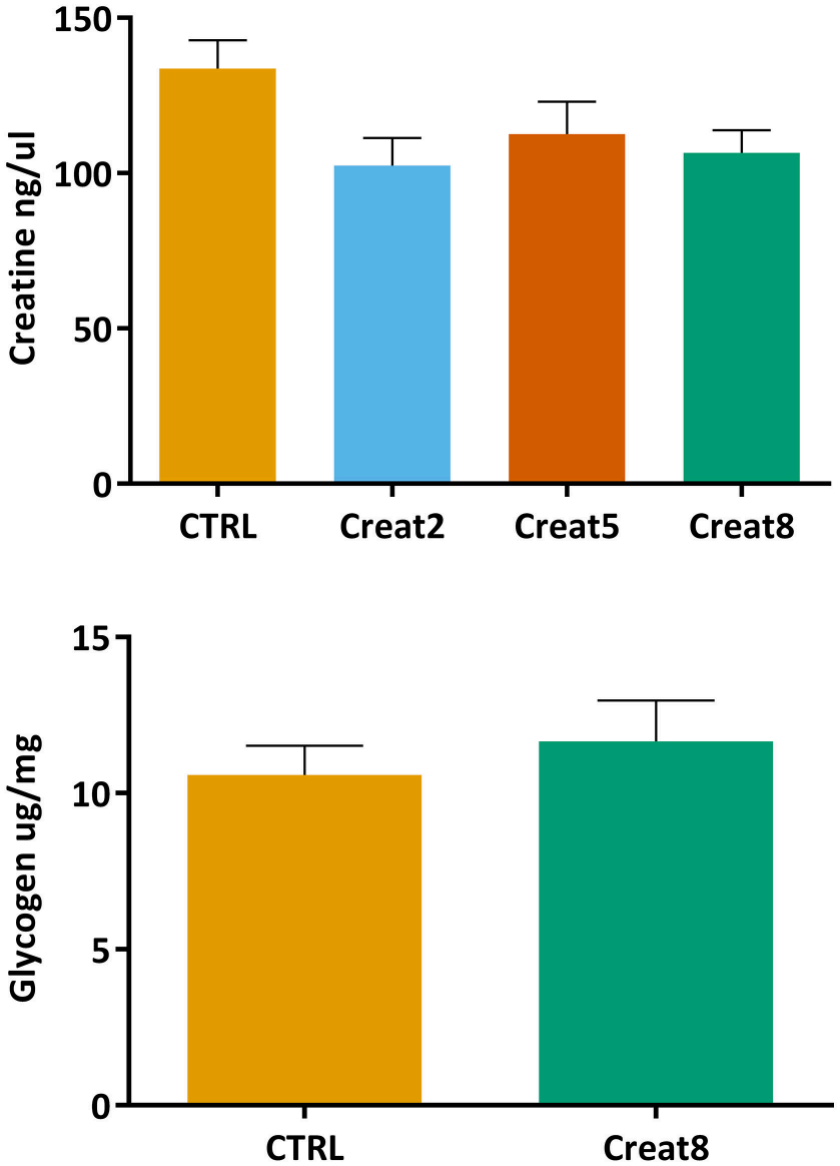
Creatine (ng/μl) concentration in muscle and glycogen (μg/mg) concentration of lyophilized muscle of gilthead seabream after 69 days of trial. Values are means (*n* = 9) and error bars represent standard error of the mean (SEM). No significant differences were detected (one-way ANOVA, *p* > 0.05 and Student's *T*-test, *p* > 0.05).

Creatine is naturally present in fish muscle (200–700 mg/100 g; Oehlenschläger, 2014), nevertheless it is important to address whether fish diet supplementation with creatine would lead to accumulation of this compound in the muscle. Using a commercially available colorimetric assay, we did not obtain significant differences between conditions (Figure 4). With these results we show that diet creatine supplementation in fish, up to 8%, does not result in an accumulation of this supplement in the muscle of these vertebrates, after 69 days of feeding. Not having similar research regarding this issue in fish, we can only speculate that creatine is entirely processed on daily basis fish activity. It should be noticed that the muscle of fish fed the control diet showed insignificant higher expression of creatine when compared to supplemented diets. Such fact suggests that a longer trial would accentuate the differences reflecting the effect of supplementation, nevertheless this implicates more driven research to confirm such indication.

The energy reserves in fish muscle were analyzed by determining the glycogen content (Figure 4). This assay was only performed using the muscle from fish fed with 0% (control) and 8% supplemented creatine diets. No significant differences were obtained between these two conditions (Student's *T*-test, *p* > 0.05), showing that creatine supplementation in the diet does not affect the energy state of the fish muscle. In case differences between the highest concentration and control samples were observed, analysis would be performed on the other supplementation percentages. To the best of our knowledge, the effect of creatine on glycogen content was correlated for the first time in fish but in humans, was shown that creatine increases glycogen storage in muscle (van Loon et al., 2004). In fish, the expression of creatine was reported to be positively correlated with energy demand (Borchel et al., 2014) which is known to be associated with higher glycogen levels (Silva et al., 2012b). The level of locomotory activity in fish is recognized to be higher than in other vertebrates. Accordingly, several research, mainly in humans, have reported that the supplementation by creatine *per se* is not sufficient to alter muscle glycogen content after intense exercise. The same research suggests that only a supplementation with creatine plus a carbohydrate is capable of sparing glycogen by decreasing the reliance on glycolysis (Robinson et al., 1999; Roschel et al., 2010). Nevertheless, in fish this compounds mix have not been tested yet and research should be conducted to draw any firm conclusions.

### Metabolic Fingerprinting by FT-IR Spectroscopy

**Figure 5:**
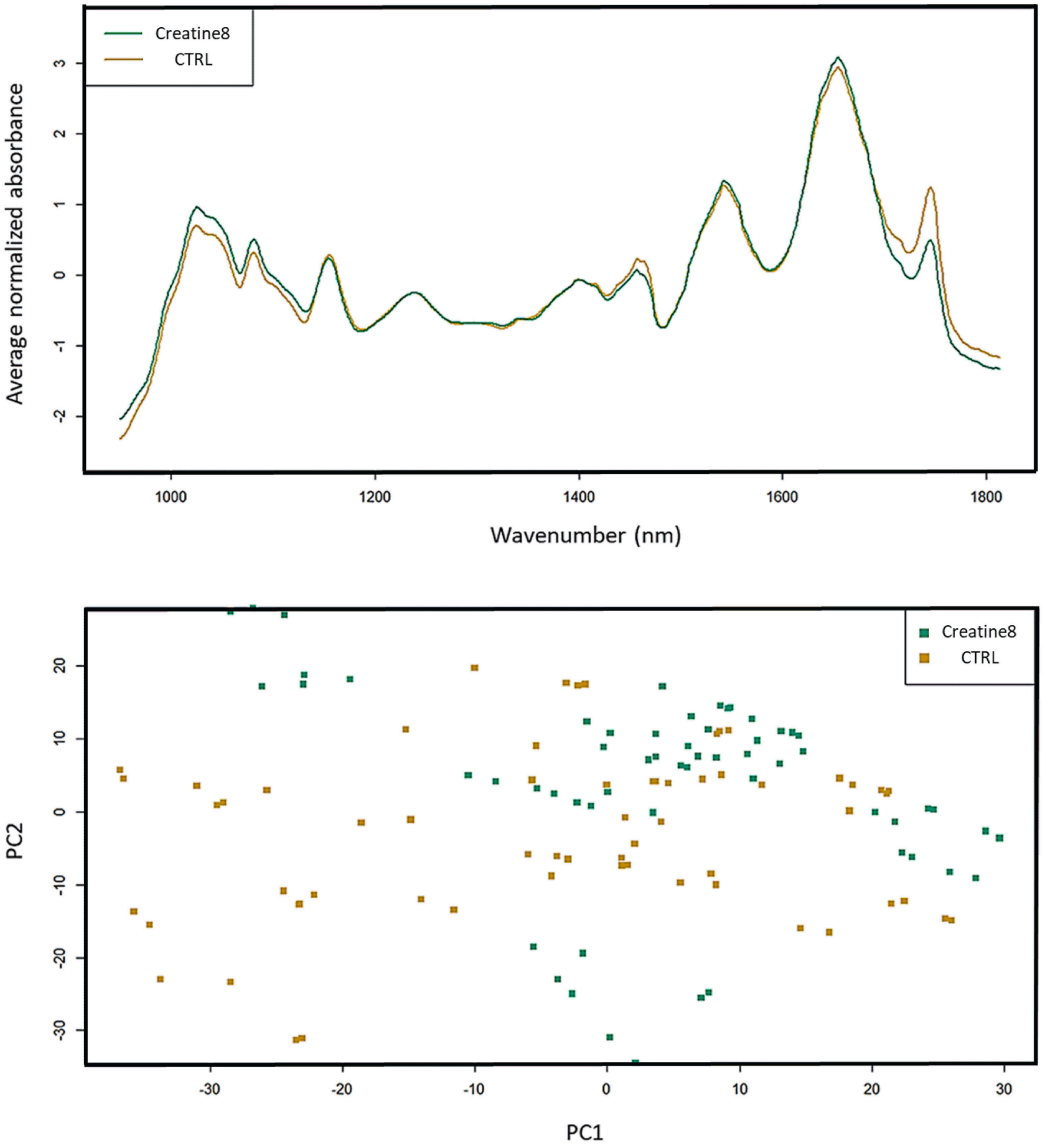
FT-IR spectra of liver of gilthead seabream. Data represents the mean (*n* = 15) for the absorption curves in the 900–1,800 cm^−1^ range. Spectra and respective PCA are shown, where control is yellow and creatine 8% is green. Differences (although not significant) are observed in the carbohydrate (1,000 cm^−1^) and lipid zone (1,450 and 1,700–1,800 cm^−1^).

Analysis of the FT-IR (Fourier-transform infrared spectroscopy) dataset shows that the biggest differences are found at 1,000 and 1,450 cm^−1^, which correspond to IR absorptions attributable to carbohydrates and lipids, respectively, (Figure 5). In the case of carbohydrates (e.g., glycogen), the feed with 8% supplementation of creatine does not alter the hepatic glycogen reserves, since fish fed with supplemented diet showed higher carbohydrate stores than the control ones. Between 1,700 and 1,800 cm^−1^ [peaks associated to lipids (triglycerides, cholesterol esters and fatty acids)] it seems like that control fish presents higher lipid reserve than fish supplemented with creatine. Several researches have been conducted in mammals showing that creatine supplementation does improve glucose tolerance and glycogen content, but it seems not enough to enhance the lipid profile in healthy individuals (van Loon et al., 2004; Op't Eijnde et al., 2006; Gualano et al., 2008). Also, in birds, creatine supplementation showed to be more beneficial in the energy metabolism by reducing the muscle glycolysis, rather than on its antioxidant activity (Wang X. et al., 2015). The principal component analysis (PCA) of all spectra shows the biological variability, with some control fish representing a comparable statistical profile to 8% creatine supplemented fish. This spatial representation reveals how misunderstanding are the exact mechanisms underlying creatine-dependent carbohydrates and lipids profiles. These synergic adaptations have yet to be clarified, however our results point out in the same direction as the prementioned studies. Notwithstanding the lack of evidences regarding the metabolic fingerprinting we would suggest further studies to investigate any indirect effect of creatine supplementation on the lipid and carbohydrate profile as a result of individuals being able to catabolize better the food nutrients. Such research could have important repercussions for the augment of farmed fish performance and aquaculture productivity.

### Muscle Quality Analysis

**Figure 6:**
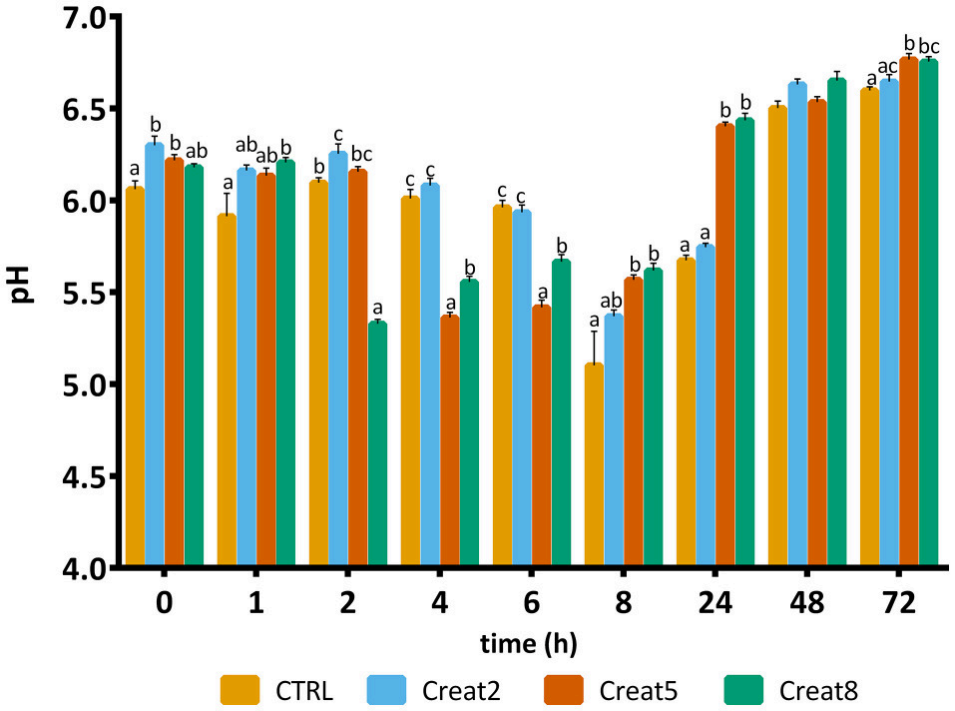
pH value of muscle of gilthead seabream fed supplemented diets with creatine. Values are means (*n* = 9) and error bars represent standard error of the mean (SEM). Different letters represent significant differences (one-way ANOVA followed by *post-hoc* Tukey, *p* < 0.05). Time is set in hours.

The quality of fish flesh is often seen by the structure and muscle quality parameters, among others like properties of skin, eyes, gills, and mucus. Textural analysis, provide important parameters of muscle quality, which are indirectly correlated with pH and *rigor mortis* (downstream indicators of the energetic status of muscle) (Silva et al., 2012b). More, both *rigor mortis* and muscle pH have been used as stress indicators in several fish species (Ribas et al., 2007; Lefèvre et al., 2008; Acerete et al., 2009). pH is known to have a great effect on conformation, thermal denaturation, and rheological properties of fish muscle proteins, particularly myosin (Tadpitchayangkoon et al., 2010). Overall, the present study seems to indicate that creatine supplementation, maintains the energy reserves of the muscle with the enhancement of the creatine levels, with potential improvement of the flesh quality. The pH measured varied between 5.0 and 6.6 over a time course of 72 h as shown in Figure 6. For all the time points measured, except after 48 h, distinct pH between experimental diets were found. Control diet revealed a basal pH of 6.2 (*t* = 0) which was significantly different from the other diets. Then, immediately after slaughtering, supplemented diets seemed to improve energy reserves (even though the lack of differences between glycogen levels as pre-explained). In humans, creatine ingestion contributed for a higher rate of phosphocreatine re-synthesis and higher pH 30 s after an intensive exercise (Yquel et al., 2002) and in rats, after an intermittent exercise, creatine loading seems to spares glycogen content (Roschel et al., 2010). Throughout storage time, pH values should decrease but our data show a constant variation of pH which is not reported in other studies (Silva et al., 2012b; Matos et al., 2013). However, the authors state that despite this variability, fish are less stressed—indicated by the lower cortisol levels and expressed higher pH after 8 h of slaughtering. Also, it seems that higher the supplementation, higher the muscle energy reserves, higher the pH and faster the *rigor mortis* (at least to some extent). Such condition is known, as pre-mentioned, linked with better texture and fish muscle quality. In accordance, a previous study, reported a correlation between higher energy reserves in muscle with a higher pH at time of death (Silva et al., 2012b).

**Figure 7:**
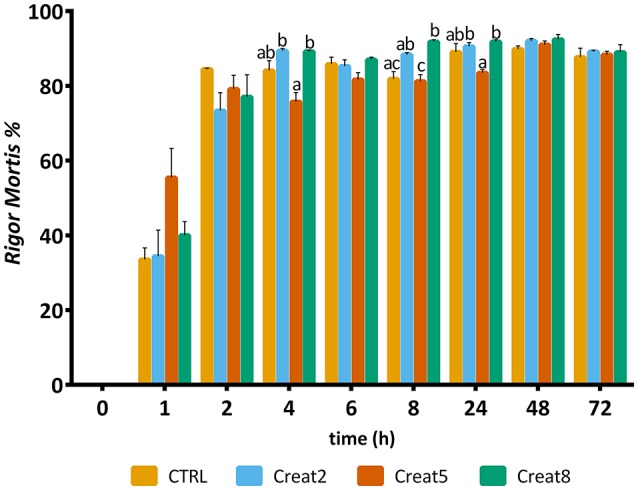
*Rigor mortis* of gilthead seabream fed supplemented diets with creatine. Values are means (*n* = 12) and error bars represent standard error of the mean (SEM). Different letters represent significant differences (one-way ANOVA followed by *post-hoc* Tukey, *p* < 0.05). Time is set in hours.

Regarding *rigor mortis* outputs, after 6 h, the majority of the fish showed such condition value higher than 80% (Figure 7). Gilthead seabream with creatine supplementation shows a typical evolution of *rigor mortis*, although some slight differences can be observed after 4, 8, and 24 h for the 5% supplementation showing less *rigor* compared to the other creatine supplementation concentrations (one-way ANOVA followed by *post-hoc* Tukey, *p* < 0.05). The reasons for such high “resistance” to *rigor* of gilthead seabream, are unknown by the authors and need to be further investigated. After 8 h, a significant difference was observed for the 8% supplementation showing more *rigor* than the control diet (one-way ANOVA followed by *post-hoc* Tukey, *p* < 0.05). Similar studies with higher energy reserves in muscle due to glycogen supplementation showed that the *rigor* status of fish have a similar pattern regarding the *rigor* continuum with our trial (Silva et al., 2012b).

Additionally, a Texture Profile Analysis (TPA) using several mechanical parameters to assess the quality of the fish flesh (see review from Cheng et al., 2014) was performed (Table 2). Briefly, hardness can be defined as the strength needed to compress the muscle between molars, adhesiveness is known as the stickiness of the muscle to a surface (palate and/or teeth), springiness can be defined as the ability of the muscle to return to the original shape, cohesiveness is known as the force needed to rupture the muscle filaments and chewiness defined as the energy needed to chew the muscle for swallowing (Hyldig and Nielsen, 2001; Careche and Barroso, 2009). In this study an effect of creatine supplementation on these parameters was not observed. No specific literature regarding texture and fish fed supplemented diets with creatine was found. Nevertheless, the available literature, mostly regarding postmortem storage and pre-slaughter stress (Suárez et al., 2005; Álvarez et al., 2008; Ayala et al., 2010), about flesh quality are *per se* contradictory, since sample preparation and sample size can easily have an effect on the repeatability, reliability and accuracy of texture parameters. Despite the care with sample preparation, the perception and measures of the texture are known to be differently affected by chemical constituents and non-homogeneous distribution of fat, moisture and collagen of fish (Cheng et al., 2014). Overall, our results suggest that a higher sample size or possibly a longer sampling period would possibly accentuate texture differences. Higher supplementation reveals a tendency to better textural properties, demonstrated by higher values of the hardness, springiness, cohesiveness, and chewiness, then suggesting evidences of enhanced muscle uptake of creatine. However, more research has to be done in understanding the role of texture and structure on fish flesh quality.

**Table 2:** Texture analysis of gilthead seabream muscle (flesh) after 69 days of trial.

**Diet**	**Hardness (N)**	**Adhesiveness (g.sec)**	**Springiness**	**Cohesiveness**	**Chewiness**
Ctrl	25.65 ± 4.60	−0.15 ± 0.06	0.63 ± 0.07	0.43 ± 0.04	6.84 ± 1.41
Creatine2	27.55 ± 5.14	−0.15 ± 0.06	0.63 ± 0.05	0.44 ± 0.04	7.59 ± 1.31
Creatine5	26.84 ± 7.25	−0.21 ± 0.15	0.62 ± 0.04	0.43 ± 0.05	7.04 ± 1.40
Creatine8	27.42 ± 4.33	−0.18 ± 0.10	0.65 ± 0.06	0.45 ± 0.03	7.94 ± 1.26

*Values are means (n = 15) ± standard deviation. No significant differences were found between the treatments for each parameter (ANOVA p > 0.05)*.

### Proteomics

**Figure 8:**
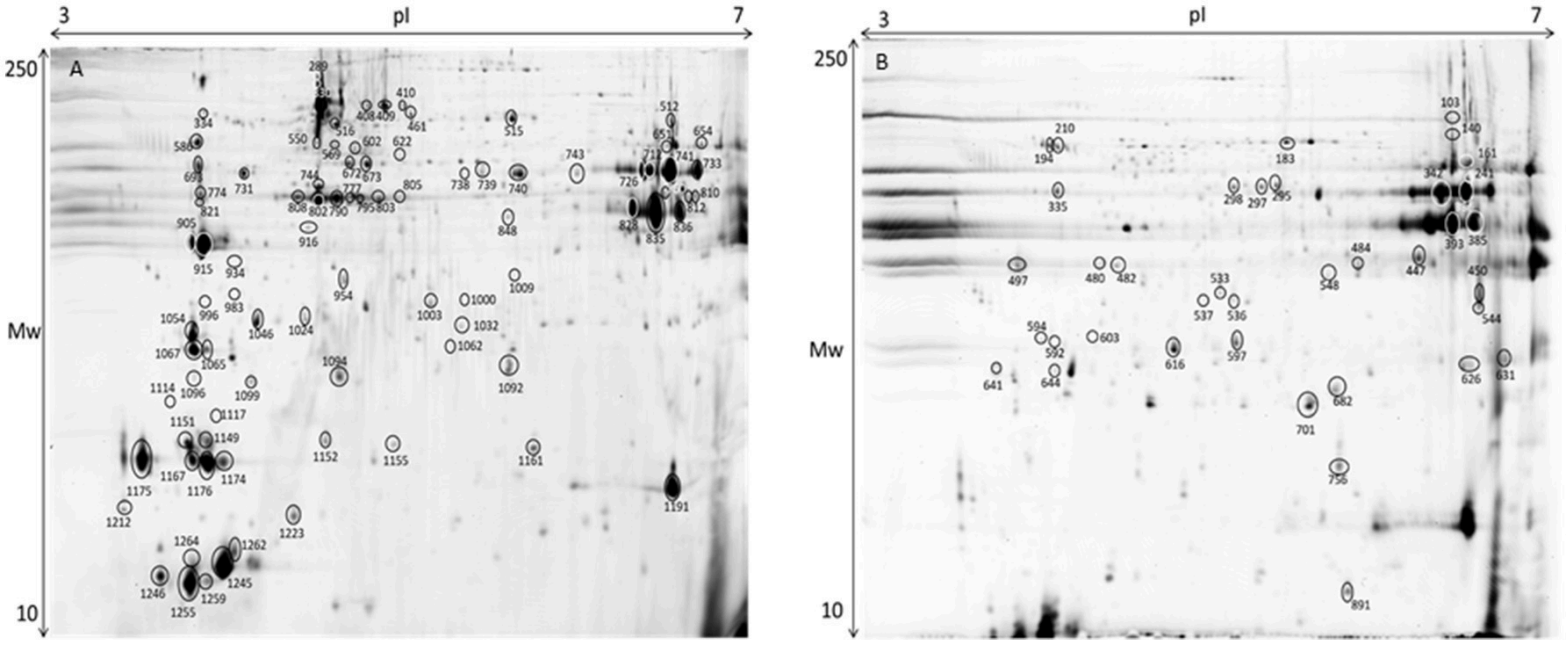
Representative 2D-DIGE gel of protein extraction of muscle of gilthead seabream in a pH range of 3–7. **(A)** Total protein extraction, **(B)** Sarcoplasmic fraction gel. Protein identifications of significantly different spots (one-way ANOVA and *post-hoc* Tukey *p* < 0.05) are shown in Table 3.

The gel images of both gilthead seabream muscle proteomes of total protein extraction and sarcoplasmic fraction are presented in Figure 8. Within the range of pH 3–7 we were able to successfully identify 127 protein spots (Table S2). Despite the wide distribution of muscle proteins on pH above 7 recognized for seabream (Martin-Perez et al., 2012), the region of interest for the majority of allergenic proteins is acid supporting the pH range selected in adequacy to the objective of this study. As so, this narrow pH was chosen to be able to get a good protein separation in the parvalbumin region, minimizing the number of overlapping spots and eliminating the usual vertical streaking of the alkaline zone. Proteins with significant differences in expression level between tested diets are shown in Table 3 with information on the protein names, accession number, molecular weight (Mw), isoelectric point (pI), score, number of peptides, coverage, fold change, false discovery rate (*q*-value) and expression for each of the identified protein spots. Regarding β-parvalbumin modulation, no effect of creatine supplementation was observed in the expression of this protein, confirming the ELISA assay results. Parvalbumins, represent the major allergens for 95% of fish-allergic patients suffering hypersensitivity to fish. Nevertheless, the cross-reactivity of parvalbumin allergenicity was shown to vary between fish species (Van Do et al., 2005; Kobayashi et al., 2016) and that allergenicity increases with parvalbumin content. The modulation of parvalbumin or its isoforms by augment of creatine concentration by oral supplementation was reported in mammals (Gallo et al., 2008; Bonilla and Moreno, 2015). The principle beyond such research intended to demonstrate that elevating the capacity for high-energy phosphate shuttling, through creatine supplementation, buffers parvalbumin expression, decreasing the allergenicity of fish. Our findings do not support such research since (1) there was no difference in creatine muscle accumulation among diets, suggesting that supplemented creatine is used on daily basis on fish activities; (2) there was no increment of energy reserves expressed by glycogen content; (3) there was no differences on parvalbumin concentration measured by ELISA. As mentioned, increasing the trial duration could refine the specific effect of creatine supplementation on fish energy metabolism. Fish could also have adapted to the daily amount of creatine given in the diet, causing protein conformational changes masked by the increase of pH as result of supplement concentration (Tadpitchayangkoon et al., 2010).

**Table 3:** Protein identification of muscle proteins in gilthead seabream.

**Biological process**	**Spot**	**Uniprot/NCBI**	**Protein name**	**Score**	**Mw T/C**	**pI T/C**	**Peptides**	**Coverage (%)**	**ANOVA**	**Tukey's Test (*q* value)**	**Fold change**	**Expression**
Energy metabolism	461	I3K2Y8	Uncharacterized protein OS *Oreochromis niloticus* [after blast 29-08-2017 myosin binding protein H (*Fundulus heteroclitus*)]	1,045	56,203/63,857	5.5/5.5	7	13	0.042	0.038	2.50	CR2>CTRL> CR8>CR5
	497	P84335	Tropomyosin alpha 1 chain OS *Liza aurata*	15,521	32,709/34,421	4.49/4.7	69	65	0.031	0.045	1.60	CTRL>CR2> CR5>CR8
	641	P82159	Myosin light chain 1 skeletal muscle isoform OS *Liza ramada*	3,451	20,054/23,673	4.34/5.0	7	36	0.010	0.042	1.95	CR8>CR2> CTRL>CR5
	651	B5DGQ7	Beta enolase OS *Salmo salar*	13,013	47,257/49,981	6.65/6.4	19	23	0.026	0.048	1.78	CR2>CTRL> CR5>CR8
	1,032	P24722	Creatine kinase testis isozyme OS *Oncorhynchus mykiss*	492	42,976/27,461	6.2/5.7	1	3	0.0005	0.002	3.27	CR5>CR2> CTRL>CR8
	1,094	gi|47221502	Unnamed protein product [*Tetraodon nigroviridis*]—after blast 29-08-2017 PREDICTED: phosphatidylethanolamine-binding protein 1 [*Xiphophorus maculatus*]	182	20,800/21,800	7.7/5.4	4	20	0.006	0.011	1.31	CR2>CTRL> CR5>CR8
Cell process /stress response	1,062	L0R689	Heat shock protein 27 Fragment OS *Gymnocephalus cernuus*	422	13,341/25,307	5.5/5.7	4	30	0.036	0.040	1.70	CTRL>CR5> CR2>CR8

*Mw, molecular weight; pI, isoelectric point; T/C, theoretical/calculated*.

Although we did not observe any significant differences in the previous discussed analysis, we found some minor effects of creatine supplementation in muscle proteome. As expected in muscle tissue proteome characterization, the majority of the identified spots are myosin, actin and tropomyosin. In case of spot 461 a blast search (http://blast.ncbi.nlm.nih.gov/) identified myosin binding protein H. This protein is up-regulated with 2% of creatine and is important for the myosin bundles in the thick filaments (Silva et al., 2012a). Moreover, beta enolase (Spot 651), a protein related with energy metabolism (Richard et al., 2016) shows to be up-regulated with 2% enrichment. Myosin light chain (spot 641) showed up-regulation in fish fed 8% creatine supplementation, supporting the idea that phosphorylation shuttling driven by creatine loading modulates contractile activity regardless the suboptimal Ca^2+^ concentrations (aka parvalbumin concentration). With 5% creatine supplementation, a creatine kinase was identified as spot 1,032. This protein belongs to the phosphotransfer network, which is important in the ATP/ADP gradients in the muscle (Silva et al., 2012a). Such specific outputs, to some extent support the findings in rainbow trout. The authors suggest that muscle seems to be independent of the import of creatine, instead it seems to produce creatine by itself (Borchel et al., 2014). Such possibility is supported by general higher locomotor activity of fish, making it energetically more beneficial to synthesize creatine at the place of usage instead of shuttling. That means, enriched creatine diets might be used for different biological processes rather than only for energetic pathways, as supported by the lack of difference in glycogen reserves. In the control fish tropomyosin alpha (spot 497), which plays an important role in muscle contraction, was up-regulated. Additionally, spot 1,062 was identified as heat shock protein 27 (hsp27). The hsp27 regulates the changing of actin filaments (Kayhan and Duman, 2010) and acts like a chaperon in case of cell damage (Schrama et al., 2017). Overall, creatine supplementation in the diets seems to influence the muscle homeostasis.

**Figure 9:**
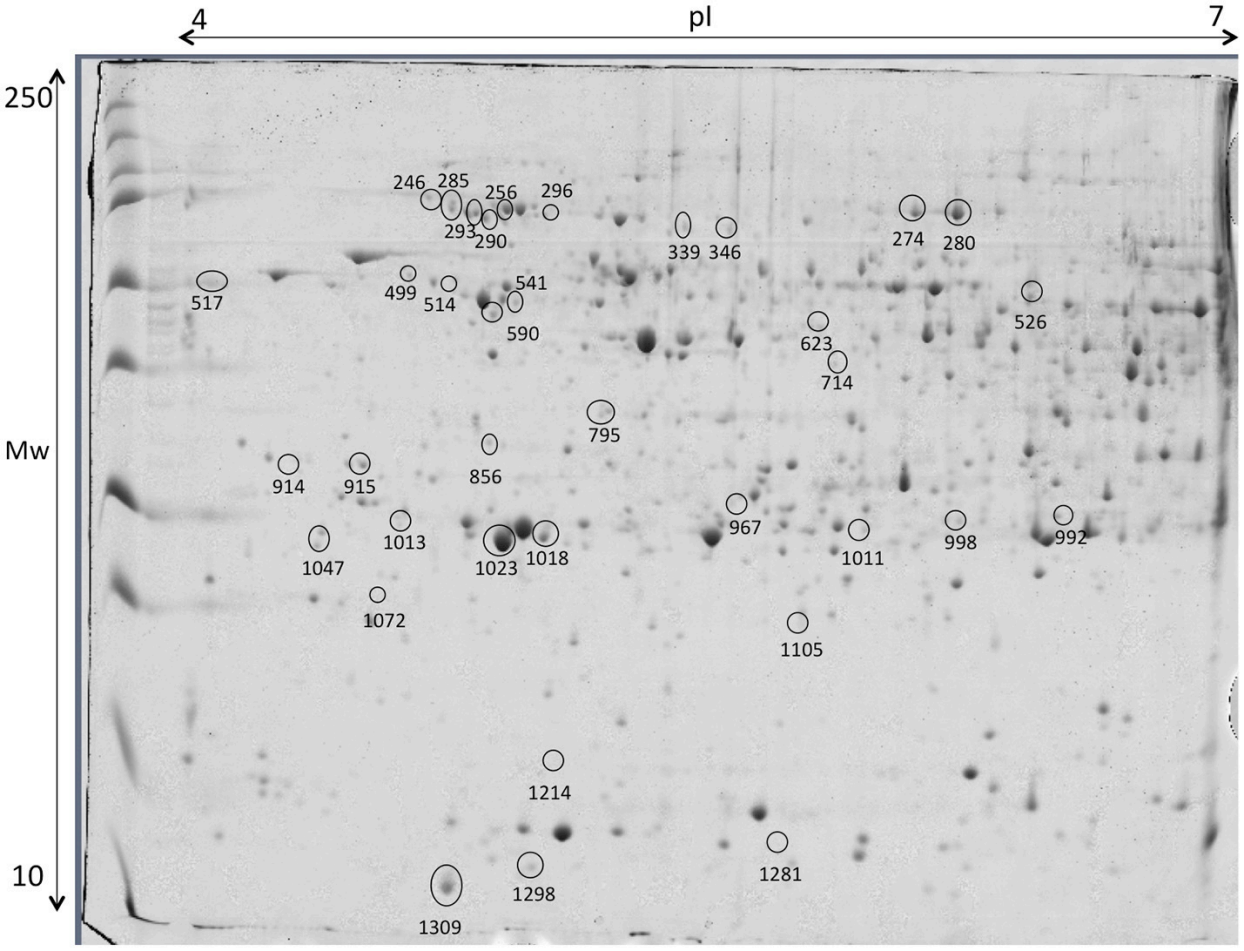
Representative 2D-DIGE gel of liver of gilthead seabream in a pH range of 4–7 on a 12.5% polyacrylamide gel. Protein identifications of significantly different spots (one-way ANOVA and *post-hoc* Tukey *p* < 0.05) are shown in Table 4.

Liver proteins were extracted to verify if the supplementation with creatine would affect the expression of proteins in this tissue, by increasing or lowering energy reserve and/or if lipid metabolism and stress proteins would be triggered. Thirty-six proteins, from a linear gradient of pH 4–7, with significant expression differences were identified, as shown in Figure 9 and Table 4, respectively. The pH selected assign the best ratio high protein coverage/good protein separation in seabream (Richard et al., 2016). Proteome analysis of control group supports the ability of these fish to activate proteins from the lipid metabolism [Apolipoprotein A (Apo-A) and 14 kDa Apo-A (Moreira et al., 2017)]. Additionally, differently expressed proteins in this experimental diet show to be involved in the immune system [Transferrin (Stafford and Belosevic, 2003); Fibrinogen beta chain (Xie et al., 2009)] and biological thermal adjustment [wap65 (Sha et al., 2008)]. Such proteins were up-regulated in control conditions, suggesting that creatine supplementation might influence the general health and biological system and lipid metabolism of these fish. Although the existence of these proteins being indicative (to some extent) of an effect of creatine supplementation, our findings could be merely arbitrary and should be founded with further applied studies. Proteins involved in cell processes and/or stress responses was shown to be up-regulated in all conditions. Nevertheless, 9 of 11 differentially expressed proteins between experimental conditions were up-regulated in liver of fish fed with creatine supplementation (Figure 9; Table 4). Specific examples are heat shock 70 kDa and heat shock cognate proteins (Schrama et al., 2017), Peroxiredoxin (Richard et al., 2016), chloride intracellular channel (Averaimo et al., 2010), and cytidine deaminase (Richard et al., 2016) which can indicate that creatine supplementation activates biological processes to protect against any cellular damage. Several proteins related to the cytoskeleton were up-regulated indifferently between the different diets, therefore indicating that the biological processes related with such function are triggered in all fish and not specifically due to creatine supplementation [glial fibrillary protein (spots 517 and 992), tropomyosin (spots 914 and 915), intermediate filament protein (spot 541), keratin (spots 590 and 1,072), cytochrome c oxidase (spot 1,298) and beta enolase (spot 526)]. Three proteins related with divergent metabolic processes were only up-regulated in fish subjected to enriched diets (described in Table 4). To some extent, these results show that liver metabolic pathways seem to be influenced by creatine enrichment nonetheless to further understand these changes more investigation needs to be conducted.

**Table 4:** Protein identification of liver proteins in gilthead seabream.

**Metabolism**	**Spot**	**Uniprot/NCBI**	**Protein name**	**Score**	**Mw T/C**	**pI T/C**	**Peptides**	**Coverage (%)**	**ANOVA**	**Tukey's Test (*q* value)**	**Fold change**	**Expression**
Immune system	246	F8U094	Warm temperature acclimation like protein Fragment OS *Epinephelus bruneu*s	990	42,159/64,966	5.46/4.8	4	7	0.001	0.0001	2.08	CTRL>CR5> CR2>CR8
	274	F2YLA1	Transferrin OS *Sparus aurata*	31,467	74,234/63,269	5.88/5.7	36	44	0.0008	0.0004	1.62	CTRL>CR2> CR8>CR5
	280	F2YLA1	Transferrin OS *Sparus aurata*	47,042	74,234/63,269	5.88/5.8	59	69	0.02	0.0207	1.57	CTRL>CR2> CR8>CR5
	285	C0L788	Warm temperature acclimation related 65 kDa protein OS *Sparus aurata*	8,120	49,126/62,438	5.34/4.9	7	16	0.0006	0.0001	2.55	CTRL>CR2> CR8>CR5
	290	C0L788	Warm temperature acclimation related 65 kDa protein OS *Sparus aurata*	11,826	49,126/61,617	5.34/4.9	12	24	0.0003	0.0000	1.94	CTRL>CR2> CR5>CR8
	293	F8U094	Warm temperature acclimation like protein Fragment OS *Epinephelus bruneus*	2,250	42,159/62,438	5.46/4.9	8	11	0.001	0.0005	2.06	CTRL>CR2> CR8>CR5
	339	A0FJG5	Fibrinogen beta chain OS *Larimichthys crocea*	1,013	55,585/60,807	5.89/5.2	4	8	0.002	0.0012	1.88	CTRL>CR2> CR8>CR5
	346	A0FJG5	Fibrinogen beta chain OS *Larimichthys crocea*	978	55,585/60,008	5.89/5.3	6	9	0.002	0.0016	1.57	CTRL>CR8> CR2>CR5
	795	Q7ZU45	Tetratricopeptide repeat protein 25 OS *Danio rerio*	134	55,545/36,781	8.70/5.1	1	2	0.002	0.0043	2.08	CR5>CR8> CR2>CTRL
Cell process/stress response	256	Q9I8F9	Heat shock 70 kDa protein 1 OS *Oryzias latipes*	1,382	70,307/63,269	5.31/4.9	2	4	0.0009	0.0034	1.72	CR5>CR2>C TRL>CR8
	296	Q90473	Heat shock cognate 71 kDa protein OS *Danio rerio* GN hspa8	266	70,930/61,617	4.99/5.0	3	6	0.03	0.0356	1.28	CR5>CR2> CR8>CTRL
	499	Q6P3H7	Histone binding protein RBBP4 OS *Danio rerio*	946	47,621/53,272	4.56/4.7	6	30	0.002	0.0015	1.42	CTRL>CR5> CR8>CR2
	514	Q0GYP4	Trypsinogen II OS *Sparus aurata*	18,962	26,240/51,881	4.98/4.8	16	49	0.016	0.0088	1.48	CTRL>CR2> CR8>CR5
	714	G3PT17	Uncharacterized protein OS *Gasterosteus aculeatus* PE 4 SV 1 [after blast on 28-04-2017 26S proteasome non-ATPase regulatory subunit 13 (*Anoplopoma fimbria*)]	2,099	43,327/41,432	5.95/5.6	14	26	0.045	0.0369	1.17	CR8>CR2> CR5>CTRL
	856	C3KGT8	Coatomer subunit epsilon OS *Anoplopoma fimbria*	2,111	34,041/33,528	4.75/4.9	10	23	0.026	0.0427	1.32	CR8>CR2> CR5>CTRL
	967	M4AWP5	Chloride intracellular channel protein [*Xiphophorus maculatus*]	327	28,409/28,988	5.84/5.3	6	30	0.044	0.0399	1.46	CR8>CR5> CR2>CTRL
	998	Q4QY74	Chymotrypsin B like protein Fragment OS *Sparus aurata*	646	23,818/27,132	7.03/5.7	5	28	0.003	0.0061	3.25	CR8>CR2> CR5>CTRL
	1,011	Q98TJ6	Glutathione S transferase Fragment OS *Platichthys flesus*	6,467	14,570/26,077	5.65/5.6	9	28	0.049	0.0247	2.63	CR2>CR8> CR5>CTRL
	1,105	G3Q5U8	Uncharacterized protein Fragment OS *Gasterosteus aculeatus* [after blast on 28-04-2017 Peroxiredoxin-1 (*Anoplopoma fimbria*)]	523	22,120/20,014	6.6/5.2	5	29	0.047	0.0278	1.27	CR5>CR2> CTRL>CR8
	1,281	F1QSJ0	Cytidine deaminase OS *Danio rerio*	3,713	14,325/10,891	7.55/5.2	2	17	0.018	0.0238	1.58	CR8>CR2> CTRL>CR5
Cytoskeleton	517	P48677	Glial fibrillary acidic protein Fragment OS *Carassius auratus*	426	42,578/51,200	4.73/4.8	1	3	0.002	0.0020	1.93	CTRL>CR2> CR8>CR5
	526	B5DGQ7	Beta enolase OS *Salmo salar*	972	47,257/49,207	6.65/5.9	3	8	0.015	0.0103	1.44	CTRL>CR2> CR8>CR5
	541	P18520	Intermediate filament protein ON3 OS *Carassius auratus*	1,416	57,753/49,207	4.95/4.9	16	23	0.023	0.0369	1.43	CR5>CR2> CTRL>CR8
	590	Q6NWF6	Keratin type II cytoskeletal 8 OS *Danio rerio*	3,453	57,723/47,292	4.94/4.9	19	28	0.015	0.0403	1.67	CR5>CR8> CR2>CTRL
	914	Q7T3F0	Tropomyosin 4 OS *Danio rerio*	770	28,484/30,161	4.43/4.5	10	26	0.015	0.0178	1.53	CTRL>CR2> CR5>CR8
	915	P13104	Tropomyosin poa 1 chain OS *Danio rerio*	1,828	32,702/30,970	4.5/4.6	7	11	0.048	0.0426	1.21	CR8>CR2> CR5>CTRL
	992	P48677	Glial fibrillary acidic protein Fragment OS *Carassius auratus*	176	42,578/27,132	4.73/6.0	1	3	0.03	0.0494	1.14	CR8>CR2> CTRL>CR5
	1,072	W5N831	Uncharacterized protein OS *Lepisosteus oculatus* [after blast on 28-04-2017 Keratin, type I cytoskeletal 19 (*Alligator mississippiensis*)]	327	88,937/21,668	4.67/4.6	5	4	0.003	0.0023	1.78	CTRL>CR2> CR8>CR5
	1,298	P80972	Cytochrome c oxidase subunit 5A 1 mitochondrial Fragment OS *Thunnus obesus*	5,414	2,402/10,329	4.28/4.9	1	50	0.007	0.0097	1.53	CTRL>CR5> CR8>CR2
Lipid metabolism	1,018	O42175	Apolipoprotein A I OS *Sparus aurata*	22,152	29,615/26,077	5.03/5.0	31	68	0.004	0.0058	1.79	CTRL>CR5> CR2>CR8
	1,023	O42175	Apolipoprotein A I OS *Sparus aurata*	28,332	29,615/25,396	5.03/4.9	59	70	0.003	0.0081	2.12	CTRL>CR8> CR2>CR5
	1,047	Q5KSU1	Apolipoprotein A IV4 OS *Takifugu rubripes*	2,047	28,474/25,734	4.59/4.6	4	12	0.039	0.0278	1.8	CTRL>CR2> CR8>CR5
	1,309	Q4QY86	Putative uncharacterized protein OS *Sparus aurata* [after blast on 28-04-2017 14 kDa apolipoprotein (*Epinephelus bruneus*)]	12,510	15,857/9,797	5.03/4.8	9	48	0.006	0.0137	2.19	CTRL>CR8> CR2>CR5
Metabolic pathway	623	Q66I24	Argininosuccinate synthase OS *Danio rerio*	504	47,099/46,671	6.46/5.5	5	7	0.01	0.0112	1.32	CR5>CR2> CR8>CTRL
	1,013	Q1MTI4	Triosephosphate isomerase A OS *Danio rerio*	2,170	26,836/26,776	4.72/4.7	6	25	0.002	0.0023	2.03	CR2>CR5> CR8>CTRL
	1,214	G3PDP5	Uncharacterized protein OS *Gasterosteus aculeatus* [after blast on 28-04-2017 bifunctional protein GlmU-like (*Salmo salar*)]	1,661	15,732/13,637	5.34/5.0	1	9	0.016	0.0462	1.73	CR8>CR2> CR5>CTRL

*Mw, molecular weight; pI, isoelectric point; T/C, theoretical/calculated*.

## Conclusion

The findings of the present study show disparities with previous studies in mammals in which creatine loading improves muscle performance (e.g., fatigue resistance, contraction efficiency; strength gain and muscle growth). In fish, driven research is scarce but our data suggests that creatine enrichment up to 8% does not seem to have an effect in major biochemical and quality aspects of fish. We observed however that cortisol levels are lower in the highest percentage of creatine supplementation, making possible to infer about the effect of creatine enrichment on fish physiological primary response. Notwithstanding with the lack of evidences regarding the effect of creatine supplementation in fish, our comparative proteomic studies has shown proteins involved in the energy homeostasis and muscle contraction mechanisms of this tissue. In liver the majority of proteins involved in immune system, lipid metabolism, metabolic pathways and cell processes suggests to be modulated by creatine supplementation. Such fact endorses to our theory that supplemented creatine is not load directly in muscle but is rather shuttled to other biological tissues and processes. Moreover, our research does not show direct evidences pointing to a modulation of allergenicity in fish fed with creatine enriched diets. Deeper research is needed to understand the co-regulation between supplemented creatine and parvalbumin, as well as to tune creatine supplementation regarding aquaculture sustainability. Hence, it is important to refer that a proper economical study should be performed to evaluate the balance between the costs of enrichment diets and how it will improve aquaculture productivity (e.g., growth, reproductive success, welfare, muscle quality, among other). Overall, this study proves the sensibility of proteomics to detect changes in fish tissues (muscle and liver) submitted to enriched diets where no apparent changes were detected by standard biochemical and quality analysis. Proteomics is however an expensive technique and therefore not always accessible in all laboratories but being such a sensitive technology, it is an invaluable tool for an untargeted and unbiased assessment of the impact of exogeneous stimuli on fish metabolism. Such possibility enables approaches toward knowledge discovery which are less hypothesis-driven and more data-driven. Nonetheless, it should be taken in mind that the lack of information of most fish genomes databases can be the major drawback and the major reason for a low protein identification rate in fish.

## Ethics Statement

This experiment was performed by trained scientists and following the European Directive 2010/63/EU of European Parliament and of the Council of European Union on the protection of animals used for scientific purposes. CCMAR facilities and their staff are certified to house and conduct experiments with live animals (group-1 license by the Direção Geral de Veterinaria, Ministry of Agriculture, Rural Development and Fisheries of Portugal).

## Author Contributions

DS was involved in the field and laboratory work execution, data analysis, and writing of the manuscript. MC was involved in the revision and writing of the manuscript by giving scientific and editorial advice. CR and RC were involved in laboratory work execution and writing of the manuscript by giving scientific and editorial advice. AR was involved in the laboratory execution of FT-IR and writing of the manuscript by giving scientific and editorial advice. TW was involved in the laboratory work execution of mass spectrometry and writing of the manuscript by giving scientific and editorial advice. AG and CC were involved in the laboratory execution of sensorial analysis. FF was involved in statistical analysis and PR was involved in work planning and writing of the manuscript by giving scientific and editorial advice.

### Conflict of Interest Statement

The authors declare that the research was conducted in the absence of any commercial or financial relationships that could be construed as a potential conflict of interest.
