# Supplementary material for: Dietary Creatine Supplementation in Gilthead Seabream (Sparus aurata): Comparative Proteomics Analysis on Fish Allergens, Muscle Quality, and Liver
Source: Front Physiol. 2018 Dec 21;9:1844. doi: 10.3389/fphys.2018.01844 (PMC6308192; doi:10.3389/fphys.2018.01844)
Supplement: Supplementary file 1 [file Table_1.DOCX]

Table S1. Ingredients and proximate composition of the control diet

| **Ingredients** | **CTRL (%)** |
| --- | --- |
| Fishmeal LT^a^ | 10.00 |
| Fishmeal 60^b^ | 10.00 |
| Porcine blood meal | 5.00 |
| Soy protein concentrate^c^ | 10.00 |
| Wheat gluten^d^ | 10.00 |
| Corn gluten^e^ | 7.25 |
| Rise protein concentrate | 3.50 |
| Soybean meal^f^ | 10.00 |
| Rapeseed meal | 4.00 |
| Wheat meal | 12.00 |
| Fish oil^g^ | 14.50 |
| Vit&Min Premix^h^ | 0.15 |
| Soy lecithin^i^ | 2.00 |
| Antioxidant | 0.40 |
| Dicalcium phosphate^j^ | 0.50 |
| L-Lysine^k^ | 0.50 |
| DL-Methionine | 0.20 |
| Creatine (g/kg)^l^ | 0.00 |
| ***Proximate composition*** |  |
| Dry Matter (DM) (%) | 95.39 ± 0.04 |
| Crude protein (%DM) | 49.28 ± 0.14 |
| Lipid (%DM) | 20.37 ± 0.31 |
| Ash (%DM) | 8.39 ± 0.06 |
| Gross energy (kJ/g DM) | 23.43 ± 0.07 |

a Peruvian fishmeal LT: 71% crude protein, 11% crude fat, EXALMAR, Peru.

b Fish by-products meal: 540 g Kg-1 CP, 80 g kg-1 CF, COFACO, Portugal.

c Soycomil P: 65% CP, 0.7% CF, ADM, The Netherlands.

d VITEN: 85.7% CP, 1.3% CF, ROQUETTE, France.

e GLUTALYS: 61% CP, 8% CF, ROQUETTE, France.

f Solvent extracted dehulled soybean meal: 47% CP, 2.6% CF, SORGAL, Portugal.

g Henry Lamotte Oils GmbH, Germany

h PVO40.01 SPAROS standard premix for marine fish, PREMIX Lda, Portugal.

i Yelkinol AC (65% phospholipids): 750 g Kg-1 CF,ADM, The Netherlands

j Dicalcium phosphate: 18% phosphorus, 23% calcium, Fosfitalia, Italy.

k L-Lysine HCl 99%: Ajinomoto Eurolysine SAS, France

l Creatine monohydrate: Sigma-Aldrich, USA

Main ingredients were grinded (below 250μm) in a micropulverizer hammer mill Hosakawa, model #1 (Hosokawa Micron Ltd., United Kingdom). These triturated ingredients were then mixed accordingly to the target formulation in a Double-helix Mixture TGC, model 500L (TGC Extrusion, France), to attain a basal mixture (no oils were added at this stage). All diets were manufactured by extrusion (pellet size 5.0 mm) by means of a pilot-scale twin-screw extruder CLEXTRAL BC45 (Clextral, France) with a screw diameter of 55.5 mm and temperature ranging 105 – 110ºC. Upon extrusion, all batches of extruded feeds were dried in a convection oven (OP 750-EF, LTE Scientifics, United Kingdom) for 2 hours at 60ºC. After this process, pellets were left to cool at room temperature, and subsequently the creatine was mixed with fish oil fraction in concentrations (2, 5 and 8%) according to each target formulation and added under vacuum coating conditions in a Pegasus vacuum mixer (PG-10VCLAB, DINNISEN, The Netherlands) respective mixture.

| Spot  Table S2 - Protein identification of muscle proteins in gilthead seabream. Mw – molecular weight, pI – isoelectric point, T/C – theoretical/calculated, FDR – false discovery rate | Uniprot / NCBI | Protein name | Score | Mw T/C | pI T/C | Peptides | Best sequence | Coverage (%) |
| --- | --- | --- | --- | --- | --- | --- | --- | --- |
| 103 | gi\|359390897 | Muscle-type creatine kinase [*Sebastes inermis*] | 345 | 42900/141400 | 6.23/6.35 | 4 | R.GTGGVDTASVGGVFDISNADR.L | 15 |
| 140 | gi\|410910532 | Predicted: creatine kinase M-type-like isoform 1 [*Takifugu rubripes*] | 51 | 42400/132500 | 6.44/6.35 | 2 | K.SFLVWVNEEDHLR.V | 10 |
| 161 | gi\|41056111 | Phosphoglucomutase-1 [*Danio rerio*] | 351 | 61100/109200 | 5.69/6.4 | 4 | K.LSLCGEESFGTGSDHIR.E | 10 |
| 183 | gi\|327243042 | Transferrin [*Sparus aurata*] | 42 | 74200/126900 | 5.9/5.8 | 1 | K.ASSIEQYYGYAGAFR.C | 2 |
| 194 | gi\|224551742 | Warm temperature acclimation-related 65 kDa protein [*Sparus aurata*] | 345 | 49100/121600 | 5.36/4.85 | 5 | R.VHLDAITSDNAGNMYAFR.G | 11 |
| 210 | gi\|224551742 | Warm temperature acclimation-related 65 kDa protein [*Sparus aurata*] | 404 | 49100/119000 | 5.36/4.9 | 7 | K.ELHSEVDAVFTYQDHLYMIK.D | 17 |
| 241 | gi\|31322099 | Creatine kinase muscle isoform 2 [*Chaenocephalus aceratus*] | 328 | 42700/114000 | 6.47/6.4 | 3 | K.TFLVWVNEEDHLR.V | 14 |
| 289 | gi\|1351868 | Actin, alpha skeletal muscle | 571 | 41900/107800 | 5.11/5.3 | 7 | K.DLYANNVLSGGTTMYPGIADR.M | 26 |
| 295 | gi\|323650066 | Mitochondrial aldehyde dehydrogenase [*Perca flavescens*] | 43 | 48200/98000 | 5.24/5.75 | 1 | K.TIPIDGDYFCYTR.H | 3 |
| 297 | gi\|47217288 | Unnamed protein product [*Tetraodon nigroviridis*] (after blast on 27-04-2017 PREDICTED: methylmalonate-semialdehyde dehydrogenase [acylating], mitochondrial-like [*Takifugu rubripes*]) | 76 | 54500/98000 | 6.83/5.7 | 1 | K.AISFVGSNSAGEYIYER.G | 3 |
| 298 | gi\|188036012 | Chain A, Crystal structure of seabream antiquitin and elucidation of its substrate | 200 | 55100/96000 | 5.83/5.6 | 5 | R.VTQATLAEYEETVQK.T | 15 |
| 330 | gi\|6653228 | Skeletal alpha-actin [*Sparus aurata*] | 610 | 41800/99300 | 5.17/5.3 | 8 | K.SYELPDGQVITIGNER.F | 27 |
| 334 | gi\|410060781 | Tropomyosin [*Oreochromis mossambicus*] | 239 | 32600/80100 | 4.52/4.7 | 5 | K.TIDDLEDELYAQK.L | 13 |
| 335 | gi\|410906157 | Predicted: ATP synthase subunit beta, mitochondrial-like [*Takifugu rubripes*] | 936 | 55100/88100 | 4.96/4.9 | 9 | R.DQEGQDVLLFIDNIFR.F | 27 |
| 336 | gi\|31322099 | Creatine kinase muscle isoform 2 [*Chaenocephalus aceratus*] | 174 | 42700/88100 | 6.47/6.4 | 3 | K.TFLVWVNEEDHLR.V | 9 |
| 342 | gi\|410910532 | Predicted: creatine kinase M-type-like isoform 1 [*Takifugu rubripes*] | 367 | 42400/90000 | 6.44/6.3 | 3 | R.GTGGVDTASVGGVFDISNADR.L | 12 |
| 385 | gi\|47210809 | Unnamed protein product [*Tetraodon nigroviridis*] (after blast on 27-04-2017 PREDICTED: beta-enolase [*Larimichthys crocea*]) | 263 | 47100/69500 | 5.97/6.4 | 4 | K.LAMQEFMILPVGAANFHEAMR.I | 16 |
| 393 | gi\|432941989 | Predicted: aldo-keto reductase family 1 member B10-like [*Oryzias latipes*] | 336 | 35600/66600 | 6.02/6.35 | 5 | K.AIGISNFNKEQIEAILNKPGLK.Y | 22 |
| 408 | gi\|317418695 | Myosin binding protein H [*Dicentrarchus labrax*] | 182 | 57800/82800 | 5.72/5.45 | 3 | R.RPGNFDGGVYSCK.A | 7 |
| 409 | gi\|317418695 | Myosin binding protein H [*Dicentrarchus labrax*] | 255 | 57800/82800 | 5.72/5.5 | 4 | R.QICVQGICSLEIR.R | 11 |
| 410 | gi\|348532430 | Predicted: myosin-binding protein H-like [*Oreochromis niloticus*] | 210 | 53900/82800 | 6.63/5.55 | 3 | R.QICVQGICSLEIR.K | 10 |
| 447 | gi\|47210809 | Unnamed protein product [*Tetraodon nigroviridis*] (after blast on 27-04-2017 PREDICTED: beta-enolase [*Larimichthys crocea*]) | 577 | 47100/62400 | 5.97/6.25 | 7 | K.AGYPDKIIIGMDVAASEFYR.S | 17 |
| 450 | Q1MTI4 | Triosephosphate isomerase A OS *Danio rerio* GN tpi1a PE 2 SV 1 | 13910 | 26836/33019 | 5.58/6.45 | 21 | N.A. | 32 |
| 480 | B5DGM7 | Fructose bisphosphate aldolase A OS *Salmo salar* PE 1 SV 1 | 504 | 39531/38996 | 8.3/5.0 | 2 | N.A. | 6 |
| 482 | gi\|432922703 | Predicted: protein ADP-ribosylarginine hydrolase-like [*Oryzias latipes*] | 50 | 38900/62400 | 4.95/5.2 | 1 | R.VPYNPEGTGCGAAMR.S | 4 |
| 484 | Q6AZW2 | Alcohol dehydrogenase NADP A OS *Danio rerio* GN akr1a1a PE 2 SV 2 | 551 | 36738/34902 | 7.32/6 | 1 | N.A. | 2 |
| 512 | gi\|47210809 | Unnamed protein product [*tetraodon nigroviridis*] - after blast 21-04-2017 PREDICTED: beta-enolase [*Larimichthys crocea*] | 431 | 47100/75000 | 5.97/6.4 | 6 | K.AGYPDKIIIGMDVAASEFYR.S | 20 |
| 515 | gi\|327243042 | Transferrin (*Sparus aurata*) | 294 | 74200/77500 | 5.9/5.9 | 6 | R.CLVEGAGDVAFIK.H | 10 |
| 516 | gi\|410905149 | Predicted: heat shock cognate 70 kDa protein-like [*Takifugu rubripes*] | 388 | 71200/76300 | 5.17/5.4 | 5 | K.GPAVGIDLGTTYSCVGIFQHGK.V | 11 |
| 533 | gi\|47207795 | Unnamed protein product [*Tetraodon nigroviridis*] (after blast on 27-04-2017 S-formylglutathione hydrolase [*Larimichthys crocea*]) | 73 | 29700/49300 | 5.35/5.55 | 1 | K.AGSQIPAAEHGIIIIAPDTSPR.G | 8 |
| 536 | I3KUW7 | Uncharacterized protein Fragment OS *Oreochromis niloticus* GN LOC100705384 PE 4 SV 1 (after blast on 28-04-2017 Phosphoglycolate phosphatase [*Nothobranchius rachovii*]) | 3228 | 33907/30807 | 5.55/5.5 | 3 | N.A. | 20 |
| 537 | H2TC41 | Uncharacterized protein Fragment OS *Takifugu rubripes* GN LOC101079900 PE 4 SV 1 (after blast on 28-04-2017 Electron transfer flavoprotein subunit alpha, mitochondrial [*Fundulus heteroclitus*]) | 1668 | 34642/30807 | 5.57/5.4 | 7 | N.A. | 22 |
| 544 | Q1MTI4 | Triosephosphate isomerase A OS *Danio rerio* GN tpi1a PE 2 SV 1 | 17307 | 26836/31674 | 4.72/6.45 | 38 | N.A. | 32 |
| 548 | gi\|348514003 | Predicted: fructose-1,6-bisphosphatase isozyme 2-like [*Oreochromis niloticus*] | 207 | 36700/56000 | 6.74/6.0 | 2 | R.VPFVVGSPDDVNEYLSFVK.K | 9 |
| 550 | gi\|148228513 | Actin, alpha skeletal muscle 2 [*Xenopus laevis*] | 820 | 42000/69100 | 5.1/5.3 | 8 | R.KDLYANNVLSGGTTMYPGIADR.M | 27 |
| 569 | gi\|30268605 | Skeletal alpha-actin type 2b [*Coryphaenoides armatus*] | 646 | 41900/68000 | 5.11/5.4 | 7 | K.DLYANNVLSGGTTMYPGIADR.M | 27 |
| 586 | gi\|3063940 | Slow myotomal muscle tropomyosin [*Salmo trutta*] | 278 | 32600/76300 | 4.56/4.7 | 5 | K.TIDDLEDELYAQK.L | 13 |
| 592 | A5PLK2 | Phospholysine phosphohistidine inorganic pyrophosphate phosphatase OS *Danio rerio* GN lhpp PE 2 SV 1 | 231 | 29605/27572 | 5.0/4.8 | 2 | N.A. | 7 |
| 594 | Q6UFZ3 | 14 3 3 protein gamma 1 OS *Oncorhynchus mykiss* PE 2 SV 1 | 9942 | 28378/27958 | 4.7/4.75 | 7 | N.A. | 28 |
| 597 | gi\|410921770 | Predicted: guanidinoacetate N-methyltransferase-like [*Takifugu rubripes*] | 441 | 26800/40600 | 5.74/5.6 | 7 | K.MFEETQVPHLLQAGFK.K | 20 |
| 602 | gi\|1339977 | Skeletal myosin heavy chain, partial [*Thunnus thynnus*] | 118 | 90696/52778 | 5.48/5.3 | 17 | K.KQADSVAELGEQIDNLQR.V | 35 |
| 603 | P84335 | Tropomyosin alpha 1 chain OS *Liza aurata* PE 1 SV 1 | 144 | 32709/26819 | 4.49/4.6 | 4 | N.A. | 12 |
| 616 | gi\|312840387 | Carbonic anhydrase [*Trematomus bernacchii*] | 84 | 28200/38900 | 5.64/5.45 | 2 | K.YPAELHLVHWNTK.Y | 10 |
| 622 | Q90339 | Myosin heavy chain fast skeletal muscle OS *Cyprinus carpio* PE 2 SV 2 | 856 | 221461/54234 | 5.4/5.4 | 15 | N.A. | 6 |
| 626 | O57656 | Glycerol 3 phosphate dehydrogenase NAD cytoplasmic OS *Takifugu rubripes* GN gpd1 PE 3 SV 1 | 1372 | 38053/27193 | 6.48/6.4 | 5 | N.A. | 15 |
| 631 | Q90XG0 | Triosephosphate isomerase B OS *Danio rerio* GN tpi1b PE 2 SV 1 | 13408 | 26810/26819 | 6.5/6.5 | 21 | N.A. | 33 |
| 644 | O42175 | Apolipoprotein A I OS *Sparus aurata* GN apoa1 PE 2 SV 1 | 19805 | 29615/25022 | 5.0/4.9 | 24 | N.A. | 65 |
| 654 | gi\|410921908 | Predicted: phosphoglucomutase-1-like [*Takifugu rubripes*] | 256 | 61000/69100 | 6.31/6.55 | 3 | K.LSLCGEESFGTGSDHIR.E | 10 |
| 672 | gi\|348515631 | Predicted: desmin-like [*Oreochromis niloticus*] | 206 | 54100/63600 | 5.2/5.4 | 4 | R.LQEEIHQKEEAENNLSAFR.A | 14 |
| 673 | gi\|348515631 | Predicted: desmin-like [*Oreochromis niloticus*] | 254 | 54100/61600 | 5.2/5.45 | 3 | K.VSDLNQAVNKNNDALR.Q | 10 |
| 682 | gi\|222088001 | Adenylate kinase 1-2 [*Epinephelus coiodes*] | 319 | 21200/31400 | 8.77/5.95 | 4 | K.ATEPVIAFYEGR.G | 24 |
| 693 | gi\|3063940 | Slow myotomal muscle tropomyosin [*Salmo trutta*] | 214 | 32600/71400 | 4.56/4.7 | 3 | K.TIDDLEDELYAQK.L | 13 |
| 701 | gi\|348534170 | Predicted: protein DJ-1-like isoform 1 [*Oreochromis niloticus*] | 353 | 19800/26400 | 6.11/5.8 | 4 | K.QGPYDVVLLPGGMPGAQNLAESPAVK.E | 32 |
| 712 | gi\|47210809 | Unnamed protein product [*tetraodon nigroviridis*] - after blast 21-04-2017 PREDICTED: beta-enolase [*Larimichthys crocea*] | 610 | 47100/60600 | 5.97/6.35 | 7 | K.AGYPDKIIIGMDVAASEFYR.S | 22 |
| 726 | gi\|47210809 | Unnamed protein product [*tetraodon nigroviridis*] - after blast 21-04-2017 PREDICTED: beta-enolase [*Larimichthys crocea*] | 699 | 47100/59600 | 5.97/6.3 | 7 | R.AAVPSGASTGVHEALELR.D | 17 |
| 731 | gi\|66267496 | Zgc: 111961 [*Danio rerio*] | 592 | 55000/58600 | 5.13/4.9 | 9 | R.LVLEVAQHLGENTVR.T | 28 |
| 733 | gi\|47210809 | Unnamed protein product [*tetraodon nigroviridis*] - after blast 21-04-2017 PREDICTED: beta-enolase [*Larimichthys crocea*] | 573 | 47100/60600 | 5.97/6.5 | 6 | R.AAVPSGASTGVHEALELR.D | 17 |
| 738 | gi\|939317735 | PREDICTED: myosin heavy chain, fast skeletal muscle-like, partial [*Maylandia zebra*] | 112 | 47917/47333 | 5.51/5.6 | 10 | R.NSQRVIDSMQSTLDAEVR.S | 48 |
| 739 | gi\|768961769 | PREDICTED: myosin heavy chain, fast skeletal muscle-like, partial [*Takifugu rubripes*] | 89 | 118546/47981 | 5.16/5.6 | 14 | K.KDIDDLELTLAK.V | 27 |
| 740 | gi\|348514660 | Predicted: alpha-enolase-like isoform 1 [*Oreochromis niloticus*] | 588 | 47200/58600 | 5.97/5.9 | 7 | R.AAVPSGASTGIYEALELR.D | 21 |
| 741 | gi\|47210809 | Unnamed protein product [*tetraodon nigroviridis*] - after blast 21-04-2017 PREDICTED: beta-enolase [*Larimichthys crocea*] | 657 | 47100/59600 | 5.97/6.4 | 6 | R.AAVPSGASTGVHEALELR.D | 17 |
| 743 | Q4S1B0 | Pyruvate kinase Fragment OS *Tetraodon nigroviridis* GN GSTENG00025632001 PE 3 SV 1 | 3153 | 58040/46692 | 7.5/6 | 18 | N.A. | 23 |
| 744 | gi\|70778800 | Actin, alpha skeletal muscle [*Danio rerio*] | 821 | 41900/55800 | 5.18/5.3 | 8 | K.DLYANNVLSGGTTMYPGIADR.M | 27 |
| 756 | gi\|45387807 | Cofilin-2 [*Danio rerio*] | 217 | 18600/16800 | 8.85/5.95 | 3 | R.YGLYDATYETK.E | 18 |
| 760 | gi\|359390897 | Muscle-type creatine kinase [*Sebastes inemis*] | 507 | 42900/48100 | 6.23/6.4 | 3 | K.RGTGGVDTASVGGVFDISNADR.L | 15 |
| 774 | gi\|335955228 | Tropomyosin [*Epinephelus bruneus*] | 133 | 21800/54000 | 4.61/4.7 | 2 | R.KLVIIEGDLER.T | 13 |
| 777 | gi\|94469901 | Fast/white muscle troponin T larval isoform [*Sparus aurata*] | 339 | 27300/52200 | 10.04/5.4 | 5 | K.SALSSMGSNYSSHLQR.A | 24 |
| 790 | gi\|225716056 | Actin, alpha cardiac [*Esox lucius*] | 847 | 41900/52200 | 5.11/5.4 | 8 | R.KDLYANNVLSGGTTMYPGIADR.M | 27 |
| 795 | gi\|225716056 | Actin, alpha cardiac [*Esox lucius*] | 618 | 41900/52200 | 5.11/5.45 | 7 | K.SYELPDGQVITIGNER.F | 27 |
| 802 | gi\|70778800 | Actin, alpha skeletal muscle [*Danio rerio*] | 858 | 41900/52200 | 5.18/5.3 | 8 | K.DLYANNVLSGGTTMYPGIADR.M | 27 |
| 803 | gi\|94469899 | Fast/white muscle troponin T adult isoform [*Sparus aurata*] | 436 | 27700/51400 | 9.91/5.45 | 5 | K.SALSSMGSNYSSHLQR.A | 19 |
| 805 | gi\|1020396140 | PREDICTED: homeobox protein cut-like 1 isoform X1 [*Sinocyclocheilus grahami*] | 66 | 163214/42449 | 5.79/5.5 | 70 | R.QDETEQSRK.K | 7 |
| 808 | gi\|6653228 | Skeletal alpha-actin [*Sparus aurata*] | 537 | 41800/53100 | 5.17/5.15 | 7 | R.VAPEEHPTLLTEAPLNPK.A | 27 |
| 810 | gi\|371901819 | Creatine kinase [*Platichthys flesus*] | 557 | 43000/48900 | 6.23/6.5 | 5 | R.GTGGVDTASVGGVFDISNADR.L | 20 |
| 812 | gi\|348510129 | Predicted: nucleoside diphosphate kinase A-like [*Oreochromis niloticus*] | 414 | 17000/49700 | 7.77/6.5 | 5 | R.MMLGETNPADSKPGSIR.G | 38 |
| 821 | gi\|348525612 | Predicted: tropomodulin 4-like [*Oreochromis niloticus*] | 232 | 38100/54000 | 4.54/4.65 | 3 | K.GNSHVEFLSIAATR.S | 13 |
| 823 | gi\|410903169 | Predicted: nucleoside diphosphate kinase B-like [*Takifugu rubripes*] | 401 | 16900/13500 | 7.72/6.4 | 4 | K.YMSSGPVLAMVWEGQNIVK.L | 21 |
| 828 | gi\|29436540 | Creatine kinase, brain b [*Danio rerio*] | 41 | 42900/48100 | 5.42/6.3 | 1 | K.TFLMWVNEEDHLR.V | 3 |
| 835 | gi\|31322099 | Creatine kinase muscle isoform 2 [*Chaenocephalus aceratus*] | 547 | 42700/48100 | 6.47/6.35 | 5 | R.GTGGVDTASVGGVFDISNADR.L | 15 |
| 836 | gi\|359390897 | Muscle-type creatine kinase [*Sebastes inemis*] | 562 | 42900/48100 | 6.23/6.5 | 4 | R.LGSSEVEQVQLVVDGVK.L | 19 |
| 848 | gi\|61218043 | RecName: Full=Actin, alpha skeletal muscle | 78 | 42286/40200 | 5.23/5.7 | 2 | K.SYELPDGQVITIGNER.F | 7 |
| 891 | gi\|348504638 | Predicted: 14 kDa phosphohistidine phosphatase-like [*Oreochomis niloticus*] | 85 | 16100/10500 | 9.5/6.0 | 1 | K.IPDVEIDPEGTFK.Y | 9 |
| 905 | gi\|335955228 | Tropomyosin [*Epinephelus bruneus*] | 265 | 21800/41500 | 4.61/4.65 | 4 | R.KLVIIEGDLER.T | 20 |
| 915 | gi\|295792268 | Tropomyosin [*Epinephelus coioides*] | 386 | 32700/40800 | 4.54/4.7 | 5 | K.KATDAEGDVASLNR.R | 14 |
| 916 | gi\|29570808 | Fast muscle-specific myosin heavy chain, partial [*Danio rerio*] | 85 | 95006/37047 | 5.22/5.1 | 10 | R.QLEEKEALVSQLTR.G | 19 |
| 934 | gi\|768908418 | PREDICTED: pyruvate dehydrogenase (acetyl-transferring) kinase isozyme 2, mitochondrial-like [*Takifugu rubripes*] | 58 | 46757/31896 | 6.46/5.0 | 6 | K.NAALASAPK.H | 15 |
| 954 | gi\|197631853 | Capping protein (actin filament) muscle Z-line beta [*Salmo salar*] | 275 | 31000/35200 | 5.25/5.4 | 7 | K.ELSQVLTQR.Q | 24 |
| 983 | gi\|94469901 | Fast/white muscle troponin T larval isoform [*Sparus aurata*] | 208 | 27300/33500 | 10.04/4.85 | 5 | K.IPDGEKVDFDDIQK.K | 21 |
| 996 | gi\|1174688032 | Neuron navigator 1 isoform X7 [*Oryzias latipes*] | 65 | 187943/26723 | 8.14/4.8 | 18 | -.MSSSGLENVSK.D | 18 |
| 1000 | gi\|6653228 | Skeletal alpha-actin [*Sparus aurata*] | 125 | 42158/30206 | 5.28/5.6 | 3 | K.AGFAGDDAPR.A | 38 |
| 1003 | gi\|6653228 | Skeletal alpha-actin [S*parus aurata*] | 185 | 42185/26006 | 5.28/5.5 | 7 | K.AGFAGDDAPR.A | 54 |
| 1009 | gi\|831322033 | PREDICTED: signal-induced proliferation-associated 1-like protein 1 [*Clupea harengus*] | 64 | 182495/30206 | 6.84/5.8 | 9 | K.RPPADHTVGGSIPATDEFYTR.H | 10 |
| 1024 | gi\|6653228 | Skeletal alpha-actin [*Sparus aurata*] | 126 | 42185/24628 | 5.28/5.2 | 3 | K.AGFAGDDAPR.A | 38 |
| 1046 | gi\|6686379 | Apolipoprotein A-I | 382 | 29600/28900 | 5.07/4.9 | 7 | R.AVNQLDDPQYAEFK.T | 28 |
| 1054 | gi\|617416779 | PREDICTED: myosin light chain 1/3, skeletal muscle isoform [*Poecilia formosa*] | 222 | 20822/23007 | 4.62/4.7 | 3 | R.VFDKEGNGTVMGAELR.I | 26 |
| 1065 | gi\|7678732 | Myosin light chain 1 [*Pennahia argentata*] | 331 | 20700/25300 | 4.47/4.7 | 5 | R.VFDKEGNGTVMGAELR.I | 30 |
| 1067 | gi\|7678732 | Myosin light chain 1 [*Pennahia argentata*] | 341 | 20700/25300 | 4.47/4.65 | 5 | K.AGFEDYVEGLR.V | 29 |
| 1092 | gi\|159137835 | Peroxiredoxin 2 [*Thunnus maccoyii*] | 248 | 21800/22600 | 6.53/5.9 | 4 | R.DYGVLKEDDGIAYR.G | 24 |
| 1096 | gi\|617416779 | PREDICTED: myosin light chain 1/3, skeletal muscle isoform [*Poecilia formosa*] | 178 | 20822/19276 | 4.62/4.8 | 3 | K.IEFSADQIDDYR.E | 30 |
| 1099 | gi\|348500116 | Predicted: lactoylglutathione lyase-like [*Oreochromis niloticus*] | 201 | 20300/18200 | 4.93/4.9 | 3 | R.FSLFFLGYEDKK.E | 13 |
| 1114 | gi\|742141995 | PREDICTED: ellis-van Creveld syndrome protein isoform X1 [*Esox lucius*] | 55 | 111583/16823 | 6.38/4.6 | 9 | R.ENHPSDCVSNSKK.G | 12 |
| 1117 | gi\|974087902 | PREDICTED: protein FAM184A-like isoform X3 [*Cyprinodon variegatus*] | 79 | 136670/16062 | 5.63/4.8 | 9 | K.NDEHEEEIESLK.E | 9 |
| 1149 | gi\|542213490 | PREDICTED: tropomyosin alpha-1 chain isoform X4 [*Oreochromis niloticus*] | 67 | 32534/14682 | 4.76/4.8 | 4 | K.LDKENALDR.A | 26 |
| 1151 | gi\|326535727 | Myosin light chain 2 [*epinephelus coioides*] | 441 | 19100/15700 | 4.56/4.6 | 6 | K.NICYVITHGEEKEE. | 44 |
| 1152 | gi\|432926489 | Predicted: eukaryotic translation initiation factor 5A-1-like isoform 1 [*Oryzias latipes*] | 123 | 17200/16500 | 4.97/5.3 | 2 | K.VNLVGIDIFTNK.K | 8 |
| 1155 | gi\|6653228 | Skeletal alpha-actin [*Sparus aurata*] | 141 | 42185/14288 | 5.28/5.4 | 5 | R.AVFPSIVGRPR.H | 28 |
| 1161 | gi\|6653228 | Skeletal alpha-actin [S*parus aurata*] | 133 | 42185/13716 | 5.28/5.9 | 4 | R.GYSFVTTAER.E | 23 |
| 1167 | gi\|410902987 | Predicted: myosin regulatory light chain 2 [*Takifugu rubripes*] | 464 | 16800/14900 | 4.26/4.65 | 5 | K.DDLRDVLASMGQLNVK.N | 48 |
| 1174 | gi\|47217809 | Unnamed protein product [*Tetraodon nigroviridis*] | 438 | 16500/14500 | 4.2/4.8 | 6 | K.LKGADPEDVILSAFK.V | 41 |
| 1175 | gi\| 5852836 | Fast skeletal myosin light chain 3 [*Sparus aurata*] | 644 | 17000/15200 | 4.2/4.35 | 7 | K.EVDALQKGTYDDYVEGLR.V | 42 |
| 1176 | gi\|410902987 | Predicted: myosin regulatory light chain 2 [*Takifugu rubripes*] | 559 | 16800/14500 | 4.26/4.7 | 6 | K.LKGADPEDVILSAFK.V | 52 |
| 1191 | gi\|432889657 | Predicted: creatine kinase M-type-like [*Oryzias latipes*] | 457 | 42700/13300 | 6.34/6.4 | 4 | R.LGSSEVDQVQLVVDGVK.L | 20 |
| 1212 | gi\|5852836 | Fast skeletal myosin light chain 3 [*Sparus aurata*] | 119 | 16976/11032 | 4.36/4.4 | 2 | K.EAFGLFDR.V | 61 |
| 1223 | gi\|327358389 | Heart type fatty acid binding protein, partial [*Oryzias melastigma*] | 98 | 12600/11900 | 5.32/5.1 | 2 | K.LGEEFDETTADDRK.V | 21 |
| 1245 | gi\|261825915 | Parvalbumin [*Sparus aurata*] | 384 | 11500/9300 | 4.66/4.75 | 4 | M.PFAGLTDADVAAALDGCK.D | 43 |
| 1246 | gi\|48476449 | Parvalbumin-like protein [*Sparus aurata*] | 551 | 11500/9100 | 4.4/4.4 | 4 | K.AFLAAGDSDGDGKIGVDEFAALVK.A | 46 |
| 1255 | gi\|348502437 | Parvalbumin alpha-like [*Oreochromis niloticus*] | 237 | 11500/8700 | 4.47/4.6 | 4 | K.LFLQNFSASAR.A | 26 |
| 1259 | gi\|50953783 | Parvalbumin 2 [*Kryptolebias marmoratus*] | 446 | 11400/8700 | 4.49/4.7 | 5 | K.SGFIEEDELKLFLQNFSASAR.A | 28 |
| 1262 | gi\|6729202 | Myosin regulatory light chain [*Cyprinus carpio*] | 95 | 18998/8995 | 4.72/4.9 | 1 | K.EAFTIIDQNRDGIISK.D | 18 |
| 1264 | gi\|5852838 | Myosin light chain 2 [*Sparus aurata*] | 50 | 19180/8635 | 4.62/4.7 | 1 | K.EAFTIIDQNR.D | 11 |
